# Ergothioneine: Biosynthesis, Molecular Mechanisms, Physiological Function, and Role in Disease

**DOI:** 10.1002/mco2.70947

**Published:** 2026-09-06

**Authors:** Guojuan Yi, Dongping Yao, Chanjuan Dong, Xiao Tu, Guojian Liao, Mengfei Long, Yongmei Xie

**Affiliations:** ^1^ School of Medicine Sichuan University of Science & Engineering Zigong China; ^2^ Department of Biotherapy, State Key Laboratory of Biotherapy and Cancer Center, West China Hospital Sichuan University Chengdu China; ^3^ College of Pharmaceutical Sciences Southwest University Chongqing China; ^4^ Research and Development Center Chengdu Chuanyu Jianwei Biotechnology Co. Ltd. Chengdu China

**Keywords:** biosynthesis, enzyme engineering, ergothioneine, fermentation, metabolic engineering

## Abstract

Ergothioneine (EGT) is a diet‐derived sulfur‐containing histidine derivative with antioxidant, anti‐inflammatory, and cytoprotective properties. In humans, its absorption, tissue distribution, and long‐term retention are mainly mediated by the organic cation transporter OCTN1, which may help explain its accumulation in metabolically active and stress‐prone tissues. Increasing experimental and clinical evidence links EGT to neurodegenerative, cardiovascular, metabolic, inflammatory, and age‐related disorders, although its therapeutic value still requires confirmation in larger and longer‐term clinical studies. This review summarizes the physiological functions of EGT, its OCTN1‐dependent transport and tissue distribution, and the molecular mechanisms by which it may modulate oxidative stress, inflammation, mitochondrial function, and cellular senescence. Particular attention is given to the dual role of the OCTN1/EGT axis in aging and tumor biology. In addition, we briefly discuss current biosynthetic and biomanufacturing strategies as practical tools for obtaining high‐purity EGT for mechanistic, preclinical, and translational studies. By placing production strategies in the context of biomedical research needs, this review aims to provide a medically oriented overview of EGT biology and its potential translational applications.

## Graphical abstract

Ergothioneine is a natural thione antioxidant with significant biomedical potential. By reducing oxidative stress, inflammation, mitochondrial dysfunction, and cellular injury, ergothioneine may support interventions for aging‐related disorders, neurodegeneration, metabolic diseases, and skin protection. Synthetic biology enables sustainable and scalable ergothioneine biosynthesis, supporting its biomedical translation.

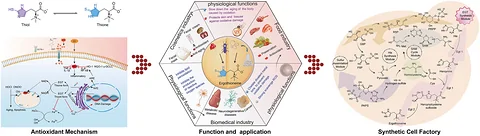

## 1 Introduction

**FIGURE 1:**
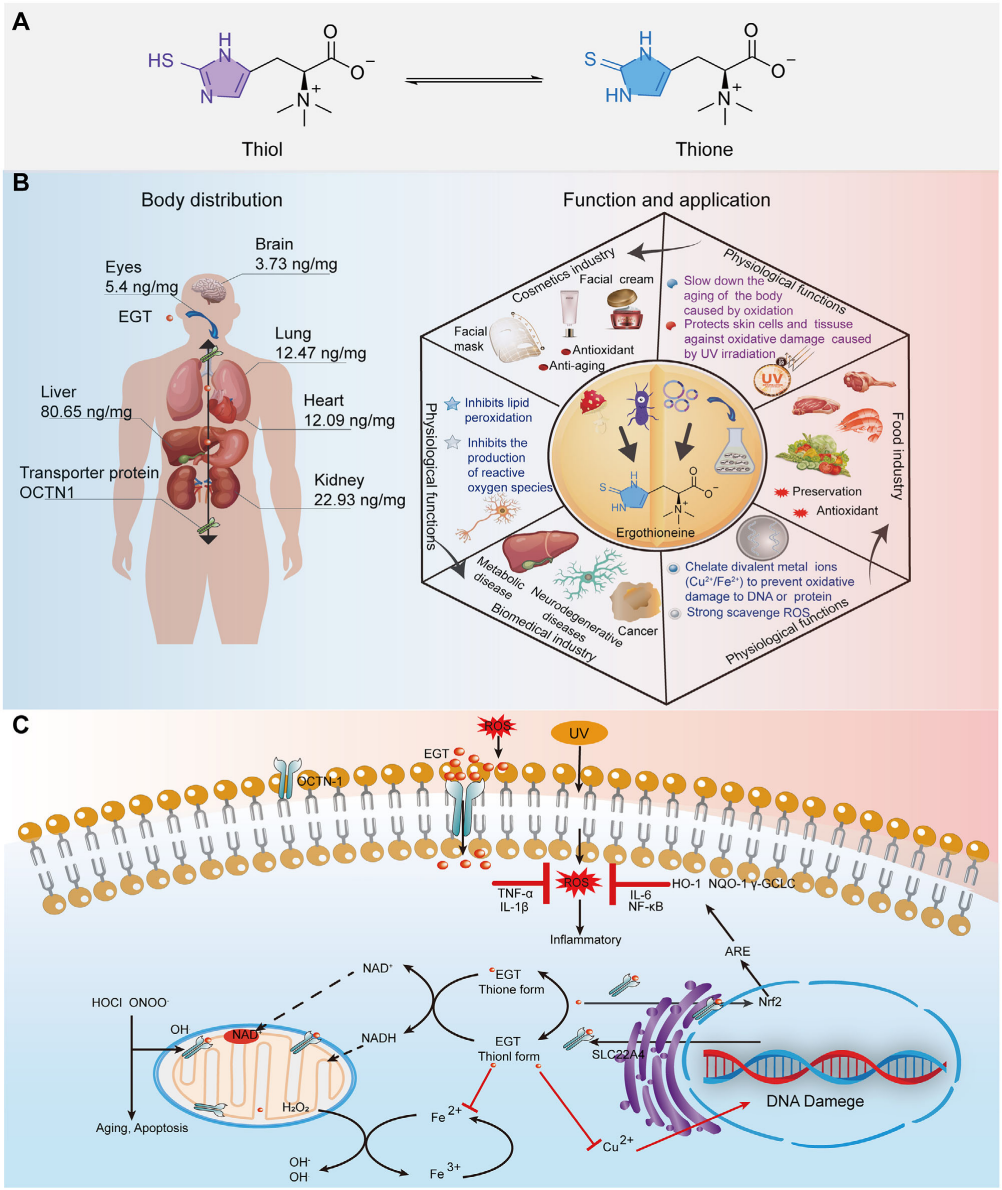
The chemical structure, antioxidant properties, and body distribution of EGT, with a focus on its multi‐industry application prospects. (A) The chemical structure of EGT. (2‐mercaptohistidine trimethylbetaine; C_9_H_15_N_3_O_2_S) and its tautomers. The thione form (right) predominates at physiological pH and thiol (left) are shown. (B) The primary organs exhibiting high EGT accumulation, ranked in descending order of EGT concentration, are as follows: liver > kidney > lung > heart > eye > brain. And EGT with various biological function was applied in food, cosmetics, and pharmaceutical fields. (C) Antioxidant mechanisms of EGT, including ROS/RNS scavenging, metal chelation (Cu^2+^, Fe^3+^), and suppression of pro‐inflammatory cytokines (TNF‐α, IL‐1β, IL‐6). OCTN1: organic cation transporter novel 1; ROS: reactive oxygen species; NQO‐1: NAD(P)H dehydrogenase, quinone 1; IL: interleukin (1β, 6); SLC22A4: solute carrier family 22, member 4; TNF: tumor necrosis factor; HO‐1: heme oxygenase 1; γ‐GCLC: glutamate‐cysteine ligase catalytic subunit; ARE: antioxidant response element; Nrf2: nuclear factor erythroid 2‐related factor 2; NAD^+^: Nicotinamide adenine dinucleotide (oxidized form); NADH: nicotinamide adenine dinucleotide (reduced form); HOCl: hypochlorous acid; ONOO^−^: peroxynitrite; OH·: hydroxyl radical; OH^−^: hydroxide ion.

Ergothioneine (EGT) is a sulfur‐containing derivative of histidine that has attracted attention because of its unusual redox stability and cytoprotective activity [1, 2]. Its molecular structure, featuring oxygen, nitrogen, and sulfur functional groups, centers on the 2‐thio‐imidazole moiety, which drives its chemical reactivity. At physiological pH, EGT predominantly exists in the thione form (Figure 1A), conferring resistance to auto‐oxidation and enhancing its antioxidant efficacy [3]. These chemical features provide a basis for its biological activity in tissues exposed to oxidative or inflammatory stress [4, 5].

Several lines of experimental evidence suggest that EGT can neutralize reactive oxygen and nitrogen species, chelate metal ions, and protect cells from oxidative damage. In vitro and in vivo studies, including mouse models, demonstrate its ability to reduce oxidative DNA damage and modulate immune responses, notably under conditions like circadian rhythm disruption [6, 7]. In human, EGT is absorbed through the organic cation transporter N1 (OCTN1) and accumulates selectively in tissues (Figure 1B). EGT concentrations are highest in the liver and erythrocytes, with substantial accumulation also observed in the intestines, semen, testis, bone marrow, kidney, spleen, lung, eye lens, and brain [8], with increased accumulation reported in some injured or inflamed tissues [9]. For instance, in Parkinson's disease models, EGT mitigates 6‐hydroxydopamine (6‐OHDA)‐induced neuronal death by suppressing excessive ROS production via OCTN1‐dependent mechanisms [10]. In addition, in murine models, EGT promotes sperm vitality and fertilization potential by facilitating capacitation and acrosome reactions in vitro [11].

Because of these properties, EGT has been explored in food, cosmetic, and biomedical settings. In the food sector, it serves as an antioxidant, color‐retention agent, and dietary supplement, enhancing the quality of premium products [5, 12, 13]. In cosmetics, EGT is prized for its safety, stability, and unique benefits, including anti‐inflammatory effects, anti‐aging properties, and non‐competitive whitening capabilities, with evidence suggesting it boosts glutathione levels in fibroblasts to promote skin health [14, 15, 16]. In biomedical research, EGT has mainly been investigated in conditions involving oxidative stress, inflammation, or mitochondrial dysfunction, including metabolic, cardiovascular, renal, hepatic, and neurodegenerative diseases [17, 18, 19, 20, 21]. The increasing demand for EGT underscores its growing significance across these industries (see Figure 1B for tissue‐specific accumulation and industrial applications).

EGT was first isolated in 1909 by Charles Tanret from the ergot fungus (*Claviceps purpurea*) in rye grains [22]. Over the past century, biochemical research has advanced, with key milestones including the crystallization of EGT from *Neurospora crassa* in 1956 and the elucidation of its biosynthetic pathways in *Mycobacterium* species through isotope tracing [22, 23]. Biosynthesis of EGT is limited to certain bacteria, fungi, and actinomycetes, leaving humans and other vertebrates reliant on dietary sources like mushrooms, grains, and animal tissues [24, 25]. Microorganisms, including aerobic *Actinomycetes*, cyanobacteria, *Schizosaccharomyces pombe*, and anaerobic green sulfur bacteria, produce EGT at modest yields (0.069–2 mg/L of culture medium) [26, 27], whereas mushrooms like *Boletus edulis* and *Pleurotus ostreatus* yield higher concentrations (0.15–7.27 mg/g dry weight) [25, 28]. Molecular studies have clarified microbial biosynthetic pathways, revealing key enzymes such as EgtB (an L‐2‐oxoglutarate‐dependent dioxygenase) and EgtA (an S‐adenosylmethionine [SAM]‐dependent methyltransferase), with variations across species indicating independent evolutionary origins [23, 29]. Despite these scientific foundations, a significant gap remains between the burgeoning clinical demand for EGT and the availability of high‐purity, scalable supplies.

Scaling EGT production remains a challenge despite its potential. Traditional methods, such as natural extraction and chemical synthesis, are limited by low yields, high costs, and environmental inefficiencies due to resource‐intensive processes and harsh reagents [12, 26]. Microbial biosynthesis offers a sustainable alternative, leveraging pathways like the EgtABCDE system in *M. smegmatis*, the Egt1/Egt2 system in *N. crassa*, and anaerobic pathways in organisms like *Bacillus stearothermophilus*, using histidine, cysteine, and methionine as precursors [30, 31]. However, constraints such as substrate specificity, enzyme efficiency, cofactor requirements, and feedback inhibition limit large‐scale production [32, 33]. Recent advances in synthetic biology and metabolic engineering are increasingly providing broadly applicable strategies to overcome bioproduction bottlenecks, thereby accelerating the sustainable translation of bioactive natural products [34]. This review first summarizes the physiological functions of EGT, with emphasis on OCTN1‐mediated uptake, tissue retention, and cytoprotective mechanisms. We then discuss the molecular pathways through which EGT may influence major disease processes, including oxidative stress, inflammation, mitochondrial dysfunction, cellular senescence, and tumor biology. Current preclinical and clinical evidence is reviewed to clarify both the translational potential and the present limitations of EGT research. Finally, because reliable access to high‐purity EGT is necessary for mechanistic and preclinical studies, we provide a concise overview of biosynthetic and production strategies.

## 2 Physiological Functions and Roles in Disease

EGT exhibits potent antioxidant properties and plays an important role in cellular homeostasis and the modulation of disease states. This section summarizes the major biological actions of EGT under physiological and pathological conditions. We first describe its OCTN1‐dependent transport, tissue retention, and cytoprotective mechanisms, and then discuss how these processes may contribute to disease modulation and clinical translation. To substantiate this framework, the review integrates supporting evidence from preclinical animal models and human clinical trials, offering a cohesive perspective on the progression of EGT from foundational biology to potential translational applications in health maintenance and disease intervention.

### 2.1 Physiological Functions, Transport Kinetics, and Cytoprotective Mechanisms

EGT is characterized by a sulfur atom appended to the 2‐position of the histidine imidazole ring and a trimethylated amino group [7, 33]. This distinctive molecular architecture predisposes EGT to predominate in the thione tautomer at physiological pH, thereby conferring enhanced stability and resistance to autoxidation relative to conventional thiols (Figure 1A). Mammals, devoid of the requisite biosynthetic machinery, procure EGT solely from dietary sources, principally fungi and select bacteria [35]. Its dietary origin and marked tissue retention have led some authors to describe EGT as a potential “stress vitamin,” although this concept still requires further clinical validation [3].

OCTN1, encoded by *SLC22A4*, is the main transporter responsible for intestinal uptake, tissue distribution, and renal retention of EGT [36]. OCTN1 exhibits high expression in intestinal epithelia, enabling efficient uptake due to its substantial substrate affinity [23]. Furthermore, it facilitates renal reabsorption, curtailing urinary elimination and characterizing EGT as an “avidly retained” compound [37]. The transport process is sodium‐dependent and electrogenic, with kinetics modulated by membrane potential and extracellular pH [38]. In response to cellular stressors, OCTN1 expression is upregulated, augmenting EGT influx to accommodate escalated demands [39]. This transport pattern helps explain why EGT is enriched in tissues with high metabolic activity or oxidative burden, such as the liver, kidney, spleen, ocular lens, brain, and erythrocytes [38, 39].

The primary physiological function of EGT is its multifaceted cytoprotective capacity, supported by robust antioxidative efficacy. EGT directly quenches a broad spectrum of ROS/RNS, specifically hydroxyl radicals (·OH), hypochlorous acid (HOCl), and peroxynitrite (ONOO^−^), which are inadequately addressed by endogenous antioxidants like glutathione [39]. This neutralization proceeds via the oxidative stress pathway, as the EGT thione moiety expedites electron transfer without generating deleterious byproducts [40]. In addition to direct scavenging, EGT sequesters divalent metal ions, effectively stopping Fenton reaction‐catalyzed ROS generation and limiting metal‐mediated nucleic acid damage [41].

EGT also orchestrates cellular stress responses through indirect modalities. By activating the Nrf2 pathway, it induces the transcription of cytoprotective genes like heme oxygenase‐1 (HO‐1) and NAD(P)H quinone oxidoreductase 1 (NQO1), thus fortifying defenses against oxidative stressors [40, 42]. Furthermore, EGT modulates sirtuin (SIRT1/SIRT6) and Poly (adp‐ribose) polymerase (PARP) activities to regulate NAD^+^ homeostasis and mitochondrial ROS levels [14, 37]. It simultaneously inhibits the NF‐κB pathway to reduce pro‐inflammatory cytokine expression and ameliorate immune‐mediated inflammation [37, 43]. Finally, by safeguarding subcellular compartments including mitochondrial genomes, polypeptides, and lipid bilayers from oxidative insult, EGT preserves cellular architecture. This protection bolsters mitochondrial bioenergetics, a key factor in metabolic equilibrium and organismal longevity (Figure 1C) [36, 39].

### 2.2 Therapeutic Applications of EGT in Disease

These cytoprotective properties have prompted studies of EGT in chronic and age‐related diseases. By engaging pathways including Nrf2 induction and ROS mitigation, EGT targets fundamental etiological mechanisms. The following subsection summarizes the evidence by disease category, with emphasis on proposed mechanisms and available experimental support.

#### 2.2.1 Neurodegenerative Diseases

EGT has been most extensively studied in neurodegenerative models, partly because OCTN1 may support its transport across the blood–brain barrier and its accumulation in vulnerable brain regions [39]. Diminished plasma EGT concentrations correlate inversely with an increased risk of mild cognitive impairment (MCI), dementia, and cognitive frailty in geriatric cohorts [44, 45]. Mechanistically, EGT attenuates excitotoxic cascades and aberrant protein oligomerization via antioxidative mechanisms, thereby preserving mitochondrial integrity and limiting neuronal apoptosis [15]. It potentiates the Nrf2/HO‐1 axis to mitigate oxidative burden in Alzheimer's and Parkinson's models while suppressing NF‐κB to reduce microglial hyperactivity and cytokine production [46, 47]. In the context of cognitive frailty, characterized by a confluence of physical debility and cognitive decline, the immunomodulatory capacity of EGT augments brain‐derived neurotrophic factor (BDNF) expression and ameliorates asymmetric dimethyl‐L‐arginine (ADMA)‐induced endothelial dysfunction [44]. Some studies also suggest that EGT may influence SIRT1/SIRT6‐related pathways, insulin/IGF‐1 signaling, and mTOR activity, although these mechanisms require further confirmation across disease models [39]. Preclinical studies demonstrate that EGT mitigates mitochondrial aberrations in senescent neurons, underscoring its prophylactic utility against neurodegeneration [15].

#### 2.2.2 Cardiovascular Diseases

EGT confers vasculoprotection by countering endothelial dysfunction, a critical early event in atherosclerosis, through the inhibition of low‐density lipoprotein (LDL) peroxidation and the protection of endothelium against ROS insults [48, 49]. Prospective cohort analyses in Scandinavian populations substantiate that elevated circulating EGT levels independently attenuate cardiovascular disease (CVD) morbidity and mortality, notably coronary artery disease [21]. Through metal sequestration and peroxynitrite neutralization, EGT represses the NF‐κB cascade to diminish pro‐inflammatory cytokine secretion and enhance vascular homeostasis [50]. It further modulates mitochondrial bioenergetics to avert ischemia‐reperfusion injury (IRI), specifically in acute myocardial infarction, by stabilizing respiratory chain complex I and attenuating ROS‐induced apoptosis [50, 51]. In the context of vascular senescence, the geroprotective attributes of EGT mitigate telomere erosion and cellular replicative exhaustion via SIRT6 induction, potentially alleviating hypertensive and cerebrovascular complications [52]. Recent investigations further demonstrate the ability of EGT to ameliorate metabolic CVD comorbidities, including the reduction of trimethylamine N‐oxide (TMAO), a microbiota‐derived atherogenic factor [53].

#### 2.2.3 Metabolic Diseases

In the context of Type 2 diabetes mellitus (T2DM) and obesity, conditions characterized by chronic inflammation and oxidative stress, EGT safeguards pancreatic β‐cells from cytokine‐triggered apoptosis via Nrf2 orchestration and ROS detoxification [42]. It attenuates metabolic dysfunction‐associated steatotic liver disease (MASLD) by limiting lipid peroxidation and hepatic inflammatory responses through the SIRT1/NF‐κB axis. This protection involves specific mechanisms including NLRP3 inflammasome repression and the inhibition of transforming growth factor‐β1 (TGF‐β1) signaling in stellate cells [53, 54]. Furthermore, polymorphisms in the *SLC22A4* gene link EGT deficiency to increased vulnerability to inflammatory bowel disease (IBD). In this setting, supplementation reinstates intestinal barrier integrity and suppresses IL‐1β/IL‐18 production via NLRP3 modulation [19, 55, 56]. In diabetic conditions, EGT enhances insulin sensitivity by activating the PI3K/AKT and FOXO pathways, thereby mitigating hyperglycemic endothelial dysfunction [15]. In addition, in obesity‐related complications, the iron‐chelating capacity of EGT prevents adipose iron overload and consequently alleviates meta‐inflammation [7, 15].

#### 2.2.4 Hematological and Other Conditions

The enrichment of EGT in erythrocytes confers resilience against oxidative hemolysis, preserving hemoglobin functionality and membrane stability. In hemolytic anemias, specifically sickle cell disease, as well as during ex vivo blood preservation, EGT diminishes cellular loss through antioxidative mechanisms [41]. Furthermore, EGT exhibits antineoplastic potential by eliciting necroptosis in neoplastic cells via mitochondrial pathways, including RIPK1/RIPK3/MLKL signaling, and by remodeling the tumor microenvironment through ROS homeostasis [57, 58, 59, 60]. Its geroprotective properties involve the regulation of IIS and mTOR pathways, which prolong healthspan by counteracting mitochondrial dysfunction and senescence [61, 62]. In hepatic pathologies extending beyond MASLD, notably IRI, the cytoprotective action of EGT entails the attenuation of Wnt5a signaling and anti‐apoptotic effects [51, 63].

### 2.3 Preclinical Animal Models and Human Clinical Evidence for EGT

Studies in rodent and invertebrate models have provided mechanistic clues to the pharmacological actions of EGT. By employing titrated dosages to achieve therapeutic tissue concentrations, these studies report protective effects in several disease models, although differences in dose, route, model system, and outcome measures limit direct comparison. The consistent engagement of key signaling pathways, notably Nrf2 activation for oxidative stress mitigation and NF‐κB/TLR4 suppression for anti‐inflammatory effects, underpins these observed benefits (Table 1). Within neurodegenerative disease models, EGT confers protection against protein misfolding, aggregation, and neuronal loss. In amyloid‐β (Aβ)‐induced Alzheimer's disease paradigms, oral administration of 5–10 mg/kg EGT significantly attenuated hippocampal Aβ plaque burden and reduced oxidative stress markers, including lipid peroxidation. This intervention restored cerebral glucose metabolism and ameliorated cognitive deficits primarily via upregulation of the Nrf2/HO‐1 axis [64, 65, 66, 67, 68]. Similarly, in MPTP‐induced Parkinson's disease models, EGT preserved dopaminergic neurons by stabilizing mitochondrial respiration and limiting microglial activation through NF‐κB inhibition [69, 70]. Beyond neurodegeneration, EGT modulated the gut–brain axis in stress‐related paradigms to alleviate REM sleep disturbances and depressive‐like behaviors by elevating BDNF levels [71, 72, 73]. Furthermore, in healthy mice, it promoted cognitive enhancement via TrkB/mTOR pathway activation [74, 75].

**TABLE 1:** Summary of preclinical animal studies on ergothioneine.

Diseases	Models	Targets/mechanisms	Major effects	Reference
Doxorubicin‐induced cardiotoxicity	C57BL‐6J mice	Inhibition of oxidative stress; chelation of Fe^2+^ preventing iron‐overload‐mediated damage; protection of mitochondrial function; decrease of proinflammatory mediators.	Protection against cardiac dysfunction and heart failure; significant protective effects on cardiac function; no interference with chemotherapeutic efficacy of doxorubicin or tumor growth.	[51]
Varicocele‐induced testicular injury	Rat model	Upregulation of HSP90AA1 expression; antioxidant protection.	Amelioration of seminiferous tubule structure disorder; decrease in testicular apoptosis; Improvement in sperm quality; Increased proliferation and reduced apoptosis in germ cells.	[65]
Alzheimer's disease	5XFAD mice	Promotion of Aβ clearance possibly by blood‐derived phagocytic macrophages and via perivascular drainage; Enhanced recruitment of IBA1(+) ve macrophages.	Enhanced Aβ clearance in neuroretina; significant reduction of Aβ immunoreactivity and of large Aβ deposits/plaques; potential neuroprotection against Aβ accumulation.	[66]
Alzheimer's disease	5XFAD mice	Reduction of amyloid plaques; reduction of oxidative stress; reduction of glucose metabolism.	Modest improvement in cognition; potential to prevent AD progression.	[67]
Alzheimer's disease	Transgenic *Caenorhabditis elegans* overexpressing human Aβ peptide	Reduction of Aβ oligomerization; antioxidant and neuroprotective properties.	Dose‐dependent reduction of Aβ oligomerization; Extension of lifespan and healthspan; delay/reduction of paralysis and premature death.	[68]
Alzheimer's disease	Mice	Inhibition of oxidative stress (prevented brain lipid peroxidation, maintained GSH/GSSG ratio, and superoxide dismutase activity); restoration of acetylcholinesterase activity; prevention of Aβ accumulation in hippocampus.	Inhibition of oxidative stress (prevented brain lipid peroxidation, maintained GSH/GSSG ratio, and superoxide dismutase activity); restoration of acetylcholinesterase activity; Prevention of Aβ accumulation in hippocampus.	[69]
Multiple system atrophy (MSA)	Human α‐Syn inducible MSA mouse model	Modification of α‐Syn species: elevation of α‐Syn monomer, significant reduction of α‐Syn dimer; reduction of α‐Syn phosphorylation in cerebral cortex; increase in human α‐Syn‐positive areas in dentate gyrus + hilus.	Significant improvement in short‐term spatial memory (nearing normal levels, associated with hippocampal activity).	[70]
Neuropsychiatric disorders	Mice (oral ergothioneine administration)	Activation of mTORC1‐S6K1 signaling (phosphorylation of S6K1 at Thr389, mTOR); Induction of neurotrophin‐4/5 (NT5/NT4/5); activation of TrkB signaling.	Promotion of neurogenesis in hippocampal dentate gyrus; Antidepressant‐like effects.	[71]
Stress‐induced anxiety and pain (psychophysical stress)	Male mice (repeated forced swim stress model)	Prevention of stress‐induced alterations in c‐Fos and FosB immunoreactivity in the PVN, NRM, and DH; dose‐dependent modulation of BDNF levels.	Prevention of anxiety‐like behaviors; reduction of pain‐like responses; improvement in cognitive recognition.	[72]
Depression	Mice	Transport across blood–brain barrier (via OCTN1); increase in brain ergothioneine concentration; promotion of neuronal differentiation.	Antidepressant‐like effects (decreased immobility time in FST and TST); achievable with human‐equivalent daily intake.	[73]
Depression/stress‐induced depressive behaviors	Rat model	Anti‐inflammatory and anti‐oxidative actions of ergothioneine; modulation via microbiota‐gut‐brain axis.	Preventive effect of oral ergothioneine on depressive behaviors (social avoidance, depression‐like sleep abnormalities, especially REM sleep disturbances)	[74]
Cognitive impairment	Male ICR mice	Activation of TrkB signaling (increased phosphorylated TrkB); involvement of neurotrophic factor receptor TrkB.	Restoration of cognitive function; reversal of impaired hippocampal neurogenesis.	[75]
Myocardial infarction	Rat model	Reduction in plasma and cellular sFlt‐1 levels; Downregulation of MCP‐1and p65; reduction in Glutaredoxin‐1 (GLRX) expression and s‐glutathionylation; inhibition of Wnt5a‐sFlt‐1 axis.	Improvement in cardiac function; reduction in infarct size; prevention of cardiac remodeling and fibrosis; reduction in cardiomyocyte death.	[76]
Cognitive enhancement	Normal ICR mice	Increase in ergothioneine concentration in plasma and hippocampus; promotion of neuronal maturation; elevation of synapsin I, neurotrophin‐3 and ‐5, phosphorylated mTOR.	Enhanced object recognition and spatial recognition memory.	[77]
Non‐alcoholic fatty liver disease (NAFLD)	Guinea pig model of NAFLD	Upregulation of transcriptional factor RUNX1 (leading to liver ergothioneine accumulation); Induction of heat shock protein 70 (corresponding to ergothioneine elevation); Fe^2+^ chelation.	No significant elevation in oxidative damage markers; suppression of oxidative damage and delay of tissue injury.	[78]
Type‐2 diabetes‐induced cardiovascular injury	Sprague–Dawley rats	Upregulation of Keap1‐Nrf2 pathway (upregulated Nrf2, HO‐1, NQO1; decreased Keap1); inhibition of Keap1‐Nrf2 complex (binding to Nrf2 active site); increased antioxidant enzymes; Reduced lipid peroxidation and inflammation.	Reduced serum triglyceride, water intake, cardiac injury biomarkers; increased body weight; protection against diabetes‐induced cardiovascular injury.	[79]
Liver fibrosis	CCl_4_‐induced liver fibrosis in mice	Promotion of nuclear translocation of Foxa3; upregulation of Hint1 transcription; elevation of Smad7; inhibition of TGFβ1‐induced HSC activation.	Suppression of hepatic stellate cell activation; Relief of CCl_4_‐induced liver fibrosis.	[80]
Iron overload‐induced hepatocellular injury	Male Wistar rats	Inhibition of NF‐κB p65 nuclear translocation; downregulation of p38 MAPK/c‐Fos phosphorylation; activation of PI3K/AKT pathway.	Alleviation of oxidative stress; improvement of liver function; attenuation of hepatocellular apoptosis/necrosis and histopathological anomalies; reduction of hepatic iron load and inflammation.	[81]
Diabetic kidney disease	Octn1 knockout and wild‐type mice (streptozotocin‐induced diabetic kidney disease model)	Promotion of oxidative stress (elevated 8‐OHdG); activation of cytoskeleton/cell adhesion pathway; induction of moesin expression.	Increased oxidative stress and exacerbated interstitial fibrosis in octn1 knockout mice; upregulated moesin expression under oxidative stress.	[82]
Type‐2 diabetes (T2D)‐associated liver toxicity	Adult male Sprague–Dawley rats	Normalized mRNA expression of SREBP‐1c, fatty acid synthase, nuclear factor kappa B, transforming growth factor β1, nuclear factor erythroid 2‐related factor 2, sirtuin‐1.	Attenuated liver hypertrophy and liver injury; reduced triglycerides, oxidative stress and inflammation; normalized related mRNA expression; reduced blood glucose and insulin resistance.	[83]
Type 2 diabetes‐induced renal damage	Male Sprague–Dawley rats	Upregulation of the Nrf2 antioxidant pathway and its downstream cytoprotective genes; inhibition of NF‐kB/TGF‐β1 mRNA expression.	Improvement of renal function; regulation of lipid metabolism; alleviation of renal structural damage; reduction of hyperglycemia and enhancement of therapeutic outcomes; enhancement of antioxidant, cytoprotective, and anti‐inflammatory effects.	[84]
Ulcerative colitis	BALB/c mice (with dextran sulfate sodium induction)	Enhancement of tight‐junction protein expression; recovery of gut mucus layer; decrease in colonic myeloperoxidase (MPO) activity; downregulation of CD4+ T cells and macrophages populations.	Prevention of body weight loss, colon shortening, increase in disease activity index, and spleen index; attenuation of gut barrier damage.	[85]
Ulcerative colitis	Rat model (with dextran sulfate sodium induction)	Strong antioxidant activity; downregulation of pro‐inflammatory factors; inhibition of TLR4/MyD88/NF‐κB signaling pathway.	Alleviation of colon length shortening and colonic pathological damage; amelioration of ulcerative colitis.	[86]
Acute lung injury	Rat model	Inhibition of inflammatory cytokines; blocking of TGF‐β/Smad2/3/Smad4 signaling; inhibition of NF‐κB activation, snail, and vimentin; upregulation of E‐cadherin.	Reduction of acute lung injury; Restoration of lung histoarchitecture (reduced congestion, cytokine infiltration, and alveolar wall thickening).	[87]
Cisplatin‐induced reproductive toxicity (testicular dysfunction)	Adult male Wistar rats	Antioxidant activity (increased SOD, GPx, CAT; decreased MDA); cytoprotectant activity.	Prevention of decreased sperm count and testosterone; reduced abnormal/dead sperm morphology; restoration of serum FSH and LH levels; protection against histopathological damage in testis; improved overall reproductive organ function.	[88]
Oxaliplatin (l‐OHP)‐induced peripheral neuropathy	Rats (repeated i.p. l‐OHP administration)	Decreased accumulation of l‐OHP in dorsal root ganglion (DRG) neurons and mitochondria (specific to OCTN1, not OCTN2)	Reduced l‐OHP accumulation in DRGs; Amelioration/prevention of mechanical allodynia	[89]
Cisplatin‐induced neurotoxicity	CBA mice (with cisplatin injection)	Inhibition of oxidative stress; restoration of acetylcholinesterase activity.	Restoration of learning and memory deficits; protection against neuronal injury and enhanced cognition.	[90]
Age‐related frailty and cognitive impairment	C57BL‐6 J wild‐type male mice	Suppression of age‐related plasma biomarkers; promotion of hippocampal neurogenesis; suppression of TDP43(brain aggregate protein) aggregation; promotion of microglial shift to M2 phenotype.	Extension of lifespan; reduced age‐related declines in weight, fat mass, movement; rescue of learning and memory impairments; promotion of healthy aging.	[91]
Aging/age‐related decline	*Drosophila melanogaster*	Regulation of autophagic activity; preservation of mitochondrial function; maintenance of GSH/GSSG ratio; enhancement of acetylcholinesterase activity.	Consistent lifespan extension; Increased climbing activity; enhanced cognition and motor function; protection against age‐related oxidative stress and decline.	[92]
Cystinuria (cystine lithiasis)	Slc7a9−/− mouse model	Increase in urinary cystine solubility; restoration of renal GSH levels and GSH metabolism; restoration of maximal mitochondrial respiratory capacity; requires internalization via transporter OCTN1.	Prevention of cystine lithiasis; no change in urinary cystine concentration or metabolic parameters.	[93]
Preeclampsia	Reduced uterine perfusion pressure rat model	Preservation of mitochondrial beta‐oxidation; increase in antioxidant‐related metabolite; reduction in inflammation‐associated metabolites.	Preservation of mitochondrial function; enhanced antioxidant levels; reduced inflammatory responses.	[94]
Preeclampsia	Reduced uterine perfusion pressure rat model	Reduction of mitochondrial‐specific hydrogen peroxide (H_2_O_2_) production; decrease in circulating antiangiogenic factor sFlt‐1.	Amelioration of hypertension; Improved fetal growth; decreased mitochondria‐specific H_2_O_2_ in kidney tissue; Reduced circulating sFlt‐1 levels.	[95]
Ischemic stroke	Rats (post‐ischemic i.c.v. infusion of ergothioneine); Mice (post‐stroke i.p. injections of ergothioneine at 70–150 mg/kg, starting 3 h after stroke)	Neuroprotection of CNS neurons.	Significant reduction of brain infarct volume from as early as 1 day post‐stroke; dose‐dependent protection; significant improvement of behavioral outcomes at 1 day after stroke.	[96]
D‐galactose‐induced aging model/learning and memory deficits	Male C57BL‐6J mice	Inhibition of oxidative stress (inhibited lipid peroxidation, maintained GSH/GSSG ratio, and superoxide dismutase activity); reduction of β‐amyloid accumulation in hippocampus; partial prevention of increased acetylcholinesterase (AChE) activity; synergistic effects with melatonin.	Improvement in active avoidance task (decreased escape latency, increased avoidance numbers) and Morris water maze performance; strongest protection against learning and memory deficits with ergothioneine + melatonin combination.	[97]
Epilepsy/PTZ‐induced seizures	*Octn1* gene knockout mice; PTZ‐induced acute seizure and kindling models	OCTN1‐mediated transport of homostachydrine (pro‐convulsant plant alkaloid substrate); reduced homostachydrine accumulation in knockout; substrate competition/inhibition by ergothioneine; modulation of epilepsy‐related genes.	Lower seizure scores in *octn1* gene knockout mice; reduced upregulation of c‐fos, Arc, BDNF post‐PTZ; homostachydrine administration increases seizure scores and gene expression; ergothioneine administration inhibits kindling progression and reduces plasma homostachydrine.	[98]
Exercise‐induced oxidative stress and inflammation	C57BL‐6J female mice	Activation of cellular NRF2 pathway; Reduction of metabolic stress, inflammation, and oxidative damage markers; higher activation of protein synthesis and satellite cells.	Longer time to exhaustion (+41.22%); decreased expression of stress and inflammation markers; reduced oxidative damage; improved muscle recovery after exercise.	[99]
Radiation‐induced gastroenteritis	BALB/c male mice	Synergistic regulation of oxidative stress; Anti‐inflammation; modulation of gut microbiota; prolonged gastrointestinal residence; inhibition of apoptosis.	Mitigation of radiation‐induced gastrointestinal tissue injury and apoptosis; reduced neutrophil infiltration and gut flora dysbiosis; protection against radiation gastroenteritis.	[100]
Exercise training adaptation	MITO‐Tag mouse	Direct binding and activation of 3‐mercaptopyruvate sulfurtransferase (MPST).	Boosting mitochondrial respiration; enhancement of exercise training performance and endurance.	[101]

*Abbreviations*: 8‐OHdG: 8‐hydroxy‐2′‐deoxyguanosine; AChE: acetylcholinesterase; AD: Alzheimer's disease; AKT: protein Kinase B; Arc: activity‐regulated cytoskeleton‐associated protein; Aβ: amyloid beta; BDNF: brain‐derived neurotrophic factor; CAT: catalase; CCl4: carbon tetrachloride; CNS: central nervous system; DH: dorsal horn; DRG: dorsal root ganglion; Foxa3: forkhead box A3; FSH: follicle‐stimulating hormone; FST: forced swim stress; FST: forced swimming test; GLRX: glutaredoxin‐1; GPx: glutathione peroxidase; GSH/GSSG: glutathione/glutathione disulfide; Hint1: histidine triad nucleotide binding protein 1; HO‐1: heme oxygenase‐1; HSC: hepatic stellate cell; HSP90AA1: Heat Shock Protein 90 Alpha Family Class A Member 1; IBA1: ionized calcium‐binding adapter molecule 1; Keap1‐Nrf2: Kelch‐like ECH‐associated protein‐1/nuclear factor erythroid‐2‐related factor‐2; LH: luteinizing hormone; l‐OHP: Oxaliplatin; MAPK: mitogen‐activated protein kinase; MCP‐1: monocyte chemoattractant protein‐1; MDA: malondialdehyde; MITO‐Tag: mitochondria‐targeted epitope tag; MPST: 3‐mercaptopyruvate sulfurtransferase; MSA: multiple system atrophy; mTOR: mammalian target of rapamycin; mTORC1: mammalian target of rapamycin complex 1; MyD88: myeloid differentiation primary response 88; NF ‐κB: nuclear factor kappa B; NQO1: NADPH quinone oxidoreductase‐1; NRF2: nuclear factor erythroid 2‐related factor 2; Nrf2: nuclear factor erythroid 2‐related factor 2; NRM: nucleus raphe magnus; OCTN1: organic cation transporter novel‐1; P13K: phosphoinositide 3‐kinase; PTZ: pentylenetetrazole; PVN: paraventricular nucleus; REM: rapid eye movement; RUNX1: runt‐related transcription factor 1; S6K1: ribosomal protein S6 kinase beta‐1;sFlt‐1: soluble Fms‐like tyrosine kinase‐1; Smad7: mothers against decapentaplegic homolog 7; SOD: superoxide dismutase; SREBP1c: sterol regulatory element‐binding protein 1c; TDP43: TAR DNA‐binding protein 43; TGFβ1: transforming growth factor β1; TLR4: toll‐like receptor 4; TrkB: tropomyosin receptor kinase B; TST: tail suspension test; α‐Syn: α‐synuclein.

Regarding cardiovascular and metabolic disorders, EGT exhibits pronounced vasculoprotective and organ‐preserving properties. In models of doxorubicin‐induced cardiotoxicity, EGT effectively chelated Fe^2+^ and diminished ROS production to preserve cardiac function without compromising chemotherapeutic efficacy [51]. Comparable protective effects occurred in IRI models where EGT stabilized mitochondrial complex I activity and suppressed inflammatory signaling via Wnt5a/NF‐κB inhibition [76]. In metabolic contexts, specifically streptozotocin‐induced diabetic rats, EGT activated Nrf2 to attenuate renal and hepatic fibrosis through the repression of NLRP3 inflammasome activity and lipid peroxidation [77, 78, 79, 80, 81, 82, 83]. In addition, in high‐fat diet‐induced non‐alcoholic fatty liver disease (NAFLD), hepatic EGT accumulation functioned as an adaptive defensive response correlating with tissue cholesterol and iron levels [78].

Extending its therapeutic scope to systemic pathologies, EGT demonstrates versatile cytoprotective capabilities. In dextran sulfate sodium (DSS)‐induced colitis, it downregulated the TLR4/MyD88/NF‐κB cascade to restore intestinal barrier integrity and reduce myeloperoxidase (MPO) activity [84, 85]. Similarly, in lipopolysaccharide (LPS)‐induced acute lung injury, EGT inhibited epithelial–mesenchymal transition (EMT) through suppression of the TGF‐β/Smad/Snail pathway [87]. Specific benefits were also noted in cisplatin‐induced neuropathy and reproductive toxicity models where EGT upregulated HSP90AA1 and restored antioxidant enzyme activities [87, 88, 89]. Moreover, EGT extended lifespan in *Drosophila* and mouse models, potentially via modulation of cholinergic neurotransmission and fatty acid β‐oxidation [90, 91]. Other applications include the improvement of fetal outcomes in preeclampsia models and the prevention of renal lithiasis in cystinuria models [92, 93, 94]. While these findings highlight the pleiotropic actions of EGT, caution is warranted when extrapolating results to humans due to potential species‐specific differences in transport, metabolism, and tissue distribution.

Building upon the extensive preclinical evidence delineating the mechanistic versatility of EGT, research has increasingly progressed toward human validation. Early‐phase studies, predominantly Phase I/II trials with modest sample sizes (Table 2), have focused on evaluating the safety and oral bioavailability of EGT at doses of 10–30 mg/day. While many applications remain confined to safety assessments and exploratory endpoints in translational contexts, the progression to larger and well‐powered clinical trials represents a critical next step for EGT‐based interventions. A pilot randomized, double‐blind, placebo‐controlled trial in older adults with MCI reported good tolerability and exploratory signals of cognitive benefit. These findings showed no alterations in clinical markers, including blood counts, renal function, or liver function. The trial also demonstrated exploratory trends toward cognitive benefits, notably improved performance on the Rey Auditory Verbal Learning Test, which suggests enhanced memory and learning abilities, and the stabilization of plasma neurofilament light chain levels, indicating a potential deceleration of neuronal damage [102]. Other completed trials targeting cognition, mood, sleep, skin health, and immune responses have similarly affirmed tolerability, although detailed efficacy outcomes remain largely unpublished. This trend reflects an emerging shift toward functionally validated and patient‐relevant applications in translational research. However, larger and longer‐term randomized controlled trials remain essential to substantiate these benefits and determine whether EGT has clinically meaningful benefits beyond safety and biomarker changes.

**TABLE 2:** Current progress in clinical trials on ergothioneine.

NCT number	Title	Phase	Status	Enrollment	Start date	Primary objective
NCT06487546	Repletion of ergothioneine in patients with kidney failure	Not applicable	Not yet recruiting	100 (estimated)	October 1, 2024	To determine whether daily ergothioneine supplementation can effectively restore blood levels of this beneficial antioxidant in dialysis patients with kidney failure.
NCT03641404	Investigating the efficacy of ergothioneine to delay cognitive decline (pilot)	Phase 1 Phase 2	Completed	19 (actual)	August 1, 2019	This randomized, double‐blind, placebo‐controlled trial aims to test whether ergothioneine supplementation can prevent or reverse cognitive decline in elderly individuals with mild cognitive impairment by leveraging its antioxidant, anti‐inflammatory, and neuroprotective properties.
NCT06886061	Effect of continuous intake of ergothioneine‐containing supplements on skin condition (EGTCLINIC)	Not applicable	Completed	70 (actual)	October 9, 2024	This was an 8‐week non‐randomized, double‐blind, placebo‐controlled clinical trial assessing the efficacy and safety of DR. ERGO Capsules (30 mg ergothioneine) on the skin condition (including brightness, elasticity, and spots) of women aged 35 to 59.
NCT05190432	Taxifolin/​ergothioneine and immune biomarkers in healthy volunteers	Not applicable	Active, not recruiting	19 (actual)	November 10, 2021	To assess how dietary Taxifolin/DHQ and/or ergothioneine alter immune function, primarily phagocytosis and oxidative burst, in healthy volunteers, particularly examining if the effects in older adults are related to their degree of immunosenescence.
NCT04556032	Effects of ergothioneine on cognition, mood, and sleep in healthy adult men and women	Not applicable	Terminated	4 (actual)	September 9, 2020	To assess the effects of ergothioneine at two doses, compared to placebo, on cognition, mood, and sleep in healthy adult men and women.
NCT04257201	Mushroom ingestion study	Not applicable	Completed	7 (actual)	August 15, 2020	To determine the effect of consuming 0, 1, or 2 servings of white button (*Agaricus bisporus*; most popular, lower ergothioneine) or yellow oyster (*Pleurotus citrinopileatus*; higher ergothioneine) mushrooms on postprandial changes in plasma levels and urinary excretions of mushroom‐specific compounds.
NCT05042674	Clinical study on rapid antioxidant protection and immune modulating effects	Not applicable	Unknown	24 (estimated)	September 20, 2021	To evaluate the acute antioxidant protection and immune effects of consuming a novel nutraceutical blend containing 25 mg of ergothioneine compared to a placebo in 24 people, using a randomized, cross‐over design over different test days with a 1‐week washout period.
NCT06846827	Cognitive, mood, anti‐inflammatory and metabolic effects of chronic oyster mushroom intervention in older adults (OYSCOG)	Not applicable	Completed	80 (actual)	October 1, 2023	To investigate the chronic effect of consuming ergothioneine‐rich *Pleurotus* oyster mushrooms (freeze‐dried powder, four times per week) on cognition and mood, while examining the underlying inflammatory, metabolic, and neurological mechanisms.
NCT06041867	*Agaricus bisporus* and influenza vaccination response	Not applicable	Completed	88 (actual)	September 14, 2023	To evaluate whether consuming *Agaricus bisporus* powder prior to influenza vaccination can improve immune function, measured by antibody response, in middle‐aged and older individuals who are overweight or obese.
NCT05896878	The effects of daily anti‐inflammatory supplementation on foundation pain index scores in chronic opiate patients	Not applicable	Terminated	1 (actual)	July 10, 2023	To evaluate the effectiveness of daily supplementation with Root. Health, a plant‐based dietary supplement, on reducing levels of 11 abnormal urine biomarkers associated with chronic pain. Biomarkers are molecules found in blood, tissues, or other body fluids that indicate normal or abnormal processes.

Abbreviation: DHQ: dihydroquercetin.

*Sources*: https://clinicaltrials.gov/. Accession date: 2026‐0620.

### 2.4 The Key Role of the OCTN1 Transporter, and Its Effects on Aging and Tumors

Although the systemic effects of EGT are often discussed in terms of antioxidant and anti‐inflammatory activity, these effects depend heavily on OCTN1‐mediated uptake and tissue retention. OCTN1 therefore provides a mechanistic link between dietary EGT intake, intracellular accumulation, and tissue‐specific biological activity. This section discusses OCTN1 first as a physiological transporter of EGT, then as a factor that may influence aging and neurodegeneration, and finally as a context‐dependent component of tumor biology.

#### 2.4.1 The Physiological Function and Kinetic Mechanics of the OCTN1 Transporter

The biological utilization and tissue retention of diet‐derived EGT are uniquely dependent upon the OCTN1, a multi‐specific polytopic transmembrane protein encoded by the *SLC22A4* gene. Discovered as a highly specialized, sodium‐dependent symporter, OCTN1 exhibits an exceptionally high substrate affinity for EGT, distinguishing it from other physiological carnitines and conventional organic cations [103]. Because humans and higher mammals lack the biosynthetic enzyme pathways required to synthesize EGT endogenously, the entire baseline systemic distribution of this compound relies on OCTN1‐mediated active transport from the intestinal lumen into the circulation, followed by its targeted uptake into peripheral organs. Unlike conventional low‐molecular‐weight antioxidants that penetrate lipid bilayers via passive diffusion, EGT requires this receptor‐driven mechanism to overcome its hydrophilic constraints and achieve therapeutic intracellular concentrations. Kinetic and sub‐cellular localization studies indicate that OCTN1 is not only localized to the plasma membrane but also selectively facilitates the trafficking of EGT into compartments highly vulnerable to metabolic stress, most notably migrating into the mitochondrial matrix and localized cellular niches characterized by high respiratory activity [17]. By mediating EGT uptake and renal retention, OCTN1 contributes to the long biological persistence of EGT in mammalian tissues. This transporter‐dependent retention is likely to be important for the cytoprotective effects attributed to EGT.

#### 2.4.2 The Strategic Impact of OCTN1 in Ageing and Neurodegeneration

The role of OCTN1 in EGT uptake is particularly relevant to aging, where oxidative stress, mitochondrial dysfunction, and chronic inflammation gradually increase. According to the classical free radical theory of ageing, the progressive, time‐dependent accumulation of ROS drives mitochondrial senescence, genomic instability, and irreversible macromolecular damage [104]. In healthy post‐mitotic tissues, particularly within the central nervous system, robust OCTN1 expression at the blood–brain barrier and on neuronal membranes allows for the continuous influx of EGT to maintain cellular redox homeostasis and mitigate neuroinflammation. However, large‐scale human clinical cohorts and epidemiological data reveal that systemic EGT levels, and by extension, the functional capacity or expression of the OCTN1 uptake pathway, decline significantly with advancing age [48]. This physiological decline correlates closely with the onset of MCI [47, 105] and the accelerated clinical pathology of Parkinson's disease [10]. When the OCTN1‐mediated transport axis is structurally or expressionally compromised, reduced OCTN1 activity may limit intracellular EGT availability and weaken EGT‐related antioxidant or anti‐inflammatory responses. Conversely, when the OCTN1 axis remains fully functional, the imported EGT successfully promotes upstream cytoprotection, suppresses the secretion of senescence‐associated secretory phenotypes (SASP), and may help delay stress‐induced senescence in neural and endothelial cells [106].

#### 2.4.3 The Pathological Subversion of OCTN1 Within the Tumor Microenvironment

However, while the targeted accumulation of EGT via OCTN1 serves as a critical defense mechanism that preserves genomic stability and prevents cellular senescence in healthy ageing tissues, the same transporter‐dependent cytoprotective mechanism may have different consequences in tumors. Malignant cells within the volatile tumor microenvironment undergo rapid, uncontrolled proliferation, a process that intrinsically generates cytotoxic levels of metabolic ROS due to altered mitochondrial energetics and hypoxia. To survive this hostile, self‐induced oxidative stress without triggering apoptosis, certain lineages of cancer cells selectively upregulate the expression of the *SLC22A4* gene, overexpressing OCTN1 to increase OCTN1‐mediated EGT uptake [48]. By co‐opting EGT's highly stable thione structure for direct radical scavenging [107] and utilizing its capacity for divalent metal ion chelation to suppress Fenton‐mediated hydroxyl radical generation [108], the overexpressed OCTN1 transporter may enhance the antioxidant capacity of tumor cells. This localized hyper‐accumulation of EGT protects the malignant mass from oxidative‐stress‐induced cell death, granting the tumor a distinct survival advantage and potentially conferring marked resistance against radiation and chemotherapeutic regimens designed to destroy cancer cells via ROS‐driven pathways [109]. Consequently, the OCTN1/EGT transport axis functions as a potential biological double‐edged sword: whereas its physiological maintenance is indispensable for neuroprotection and healthy longevity, its pathological subversion acts as a vital metabolic adaptation that facilitates tumor progression, survival, and therapeutic evasion.

In summary, the broad therapeutic spectrum of EGT in neurodegenerative, cardiovascular, and metabolic disorders is supported by substantial preclinical and emerging clinical evidence. These health‐promoting benefits establish a compelling medical rationale for its widespread application. However, the limited natural supply of EGT remains a primary bottleneck for its clinical translation.

## 3 Molecular Mechanisms of Ergothioneine Biosynthesis

EGT biosynthesis is characterized by remarkable evolutionary diversity, involving distinct enzymatic pathways optimized by different organisms to survive oxidative environments. Currently, three primary biosynthetic routes are recognized, each utilizing unique sulfur‐donors and enzymatic architectures. A broad understanding of these natural pathways is essential, as they provide the essential genetic parts for the subsequent design of high‐efficiency microbial cell factories.

### 3.1 Biosynthetic Pathways

EGT biosynthesis has evolved independently across organisms, yielding three distinct pathways: the mycobacterial, fungal, and anaerobic bacterial pathways [13, 110, 111].These pathways exhibit notable diversity in enzyme composition and substrate use while conserving core steps of histidine methylation and sulfur incorporation.

#### 3.1.1 Mycobacterial Pathway

**FIGURE 2:**
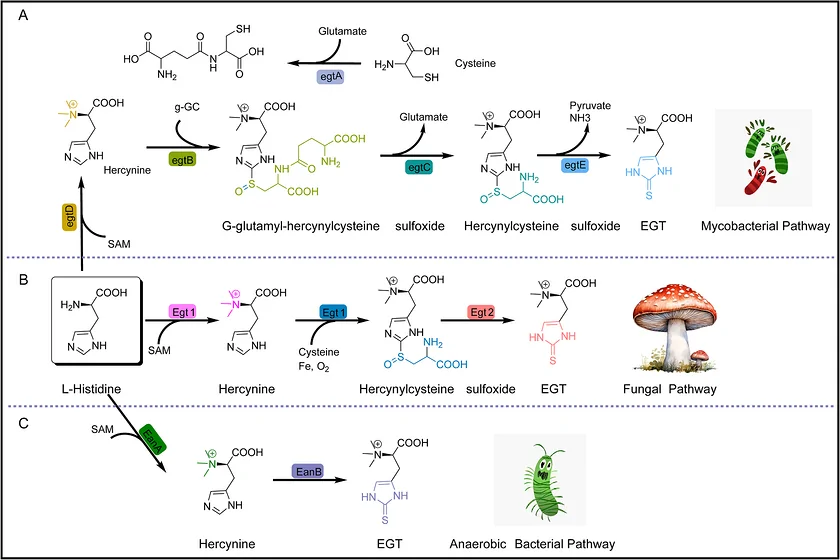
The three main EGT biosynthetic pathways. (A) The prokaryotic biosynthesis pathway, primarily observed in *Mycobacterium smegmatis*, involves five enzymatic steps catalyzed by the enzymes EgtA, EgtB, EgtC, EgtD, and EgtE. (B) The eukaryotic biosynthesis pathway, predominantly found in *N. crassa*, is driven by two enzymes, Egt1 and Egt2. (C) The anaerobic biosynthesis pathway, mainly occurring in *C. limicola*, is facilitated by the enzymes EanA and EanB. SAM: S‐adenosyl‐L‐methionine; g‐GC: γ‐glutamylcysteine; NH_3_: ammonia.

In mycobacterial, such as *M. smegmatis*, EGT biosynthesis proceeds through a five‐enzyme pathway (EgtA–EgtE), first characterized by Seebeck in 2010 [112]. The pathway begins with EgtD, a histidine methyltransferase, which uses SAM to trimethylate L‐histidine, forming hercynine (Figure 2A). Concurrently, EgtA, a γ‐glutamylcysteine synthase, combines glutamate and cysteine to produce γ‐glutamylcysteine (γ‐GC), a sulfur‐containing intermediate. EgtB, a non‐heme iron‐dependent sulfoxide synthase, then incorporates sulfur from γ‐GC into hercynine, yielding hercynyl‐γ‐glutamylcysteine sulfoxide. EgtC, a glutamine amidotransferase, removes the glutamyl group to produce hercynylcysteine sulfoxide. Finally, EgtE, a pyridoxal 5′‐phosphate (PLP)‐dependent β‐lyase, cleaves the C─S bond, releasing EGT and pyruvate. In some bacteria, such as *M. aquaticus*, an alternative pathway simplifies this process: EgtB directly combines hercynine with cysteine, bypassing EgtA and the γ‐GC intermediate, to form hercynylcysteine sulfoxide, which EgtE converts to EGT and pyruvate. This streamlined pathway may enhance production efficiency, offering potential for biotechnological applications [113].

#### 3.1.2 Fungal Pathway

In *N. crassa*, EGT biosynthesis occurs through a streamlined two‐enzyme pathway [114]. Egt1, a bifunctional enzyme (Figure 2B), combines methyltransferase and sulfoxide synthase activities to first convert L‐histidine and SAM into hercynine, then incorporates sulfur from cysteine to form S‐hercynylcysteine sulfoxide. Egt2, a PLP‐dependent cysteine desulfurase, subsequently cleaves the C─S bond of this intermediate, yielding EGT. Compared to bacterial pathways, this two‐enzyme system offers greater efficiency by bypassing the γ‐GC step, reducing competition with glutathione synthesis, a key antioxidant in eukaryotic cells. Egt1's strong substrate preference for hercynine and cysteine further enhances metabolic efficiency, while the direct conversion of Egt2 simplifies the process, facilitating potential genetic engineering applications. Notably, it has been demonstrated that expressing a single gene, *Aspergillus fumigatus* egtA, in *Saccharomyces cerevisiae* enables complete EGT biosynthesis, highlighting the adaptability of this pathway for biotechnological use [115].

#### 3.1.3 Anaerobic Bacterial Pathway

In certain anaerobic bacteria, such as *Chlorobium limicola*, EGT biosynthesis occurs through a two‐enzyme pathway involving EanA and EanB. EanA, a methyltransferase, converts L‐histidine to hercynine, while EanB, a sulfurtransferase, directly incorporates sulfur into hercynine, likely via a persulfide intermediate, to produce EGT [111] (Figure 2C). Genes homologous to *eanA* and *eanB* have been identified in other bacteria and archaea, including *Salinibacter ruber* and *Methanohalophilus mahii* [116]. These pathways underscore the evolutionary adaptability of EGT biosynthesis, reflecting the ecological and metabolic diversity of the producing organisms.

### 3.2 Key Enzymes in Biosynthetic Pathways

Recent structural and biochemical studies have deepened our understanding of the molecular mechanisms driving EGT biosynthesis, revealing the catalytic strategies and structural adaptations of its key enzymes. This conserved pathway, active in bacteria and fungi, relies on specialized enzymes‐EgtB, EgtE, Egt1, and EanB‐that orchestrate distinct steps in synthesizing EGT, a sulfur‐containing antioxidant vital for cellular protection.

One of the key steps in the EGT biosynthesis pathway, the C─S bond cleavage reaction, uses the pyridoxal 5′‐phosphate dependent C─S lyase to produce the final product L‐ergothioneine. Wei et al. presented the crystallographic structure of the ergothioneine‐biosynthesis C─S lyase EgtE from *M. smegmatis* (MsEgtE), representing the first published structure of ergothioneine‐biosynthesis C─S lyases in bacteria and showing the effects of active site residues on the enzymatic reaction [117]. The MsEgtE and the previously reported EGT biosynthesis C─S lyase Egt2 from *N. crassa* (NcEgt2) fold similarly. However, discrepancies arise in terms of substrate recognition, as observed through sequence and structure comparison of MsEgtE and NcEgt2. The structural sequence alignment of the EGT biosynthesis C─S lyase from fungi and bacteria shows clear distinctions among the recognized substrate residues, but Arg348 is a critical and highly conserved residue for substrate recognition. The α14 helix is exclusively found in the bacteria EgtE, which represent the most significant difference between bacteria EgtE and fungi Egt2, possibly resulting from the convergent evolution of bacteria and fungi.

**FIGURE 3:**
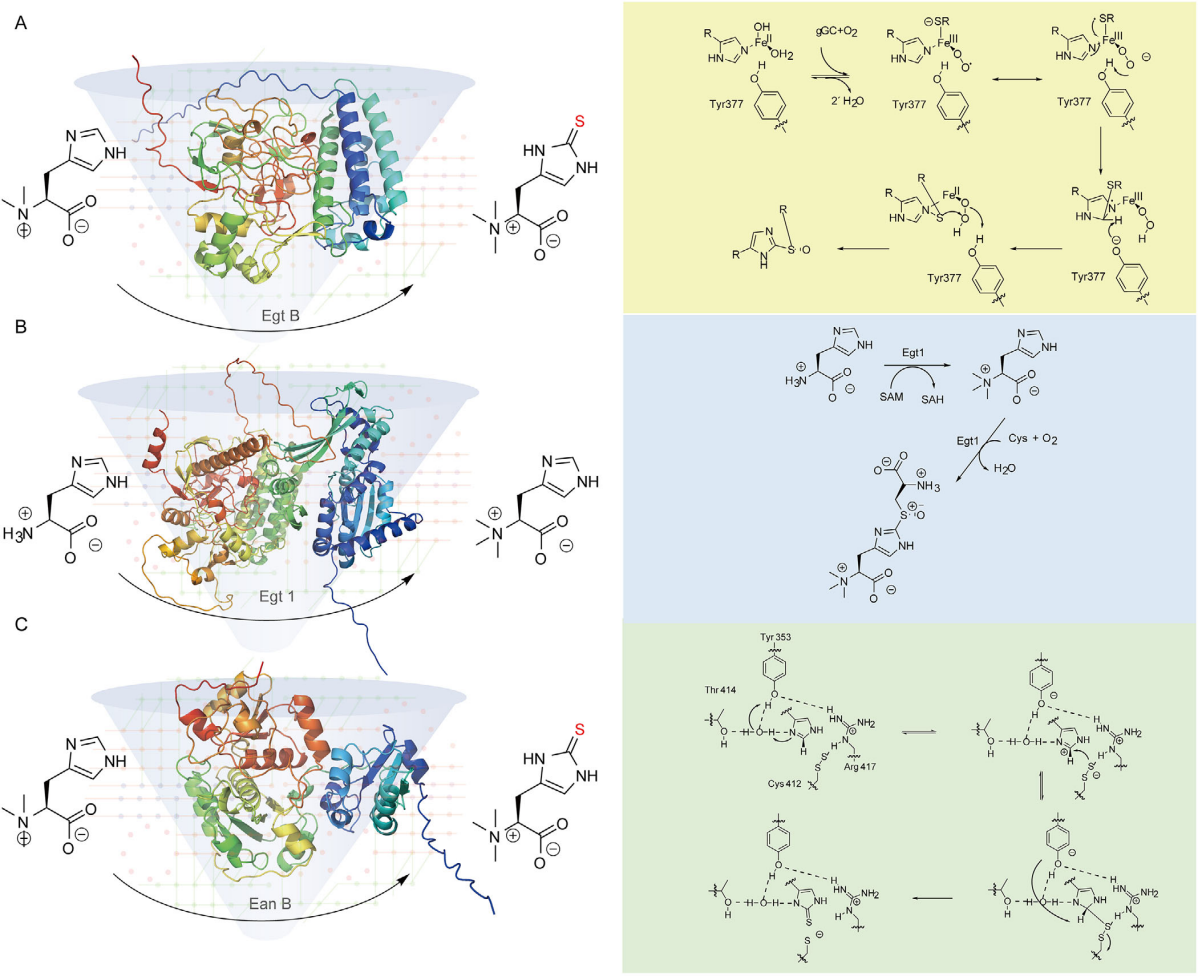
The structure and proposed mechanism of key enzymes in the EGT biosynthesis. (A) Proposed mechanism for EgtB‐catalyzed C─S bond formation and sulfoxidation; (B) Possible catalytic mechanism of Egt1 for the biosynthesis of ergothioneine, involving N‐methylation and oxidative C─S bond formation; (C) Possible catalytic mechanism of EanB for C─S bond formation coupled with S─S bond reduction.

EgtB, a mononuclear non‐heme iron enzyme, catalyzes the oxidative formation of the C─S bond, a pivotal step in EGT biosynthesis. Structural studies reveal that EgtB employs a conserved tyrosine residue (Tyr377) to oxidize of γ‐GC and transfer sulfur to hercynine, forming a sulfoxide intermediate critical to the pathway (Figure 3A). EgtB enzymes are classified into five types based on structural features [118]. For instance, Type II EgtB from *Chloracidobacterium thermophilum* preferentially uses cysteine over γ‐GC in in vitro, demonstrating substrate versatility. Notably, certain type II EgtBs from *Methylobacterium* strains enable EGT production in *Escherichia coli* through heterologous *egtB* expression, even without EgtC activity, suggesting a bypass mechanism that enhances pathway flexibility [119].

In fungi, Egt1, a bifunctional enzyme, integrates methyltransferase and sulfoxide synthase activities to streamline EGT biosynthesis (Figure 3B). Egt1 first methylates L‐histidine using SAM to produce hercynine, then incorporates sulfur from cysteine in an oxygen‐dependent reaction to yield a sulfoxide intermediate. This dual functionality reduces the need for multiple enzymes, reflecting an efficient evolutionary adaptation in fungal systems [120]. In contrast, EanB, a sulfurtransferase active in anaerobic bacteria, employs a cysteine persulfide intermediate, stabilized by residues such as Cys441 and Arg447, to transfer sulfur to hercynine under oxygen‐limited conditions [121] (Figure 3C). This mechanism underscores the adaptability of pathway to diverse ecological niches.

While these structural and mechanistic features highlight the diversity and adaptability of EGT‐biosynthetic enzymes, quantitative kinetic parameters provide essential insights into their catalytic efficiencies, substrate affinities, and potential rate‐limiting steps, enabling a more precise comparison between bacterial and fungal pathways.

In the bacterial five‐enzyme pathway, EgtD initiates EGT biosynthesis by trimethylating L‐histidine to hercynine, often acting as a rate‐limiting step due to its moderate efficiency. Kinetic studies report, *k*_cat_ = 1.3 × 10^−2^ s^−1^ and *k*_cat_/*K*_m_ = 30.8 M^−1^·s^−1^ for EgtD from *M. smegmatis*, with *K*_m_ values (422.0 ± 37.4 µM) for L‐histidine typically in the hundreds of µM range, reflecting processive methylation but low turnover typical of methyltransferases (*k*_cat_ < 1 s^−1^) [120, 121, 122]. Downstream, EgtB exhibits higher substrate affinity, with *K*_m_ for γ‐GC ≈50 µM, *k*_cat_ ≈0.15 s^−1^, and *k*_cat_/*K*_m_ ≈3000 M^−1^·s^−1^ in wild‐type variants, enabling efficient iron‐dependent C─S bond formation [123]. The C─S lyase EgtE shows strong preference for hercynylcysteine sulfoxide over thioether substrates, with *k*_cat_ = 1516 ± 27 min^−1^ and *K*_m_ = 121.2 ± 6.6 µM for the sulfoxide [31]. In contrast, fungal dual‐enzyme systems re more streamlined, with NcEgt1 (bifunctional methyltransferase‐sulfoxide synthase) favoring hercynine and L‐cysteine (*k*_obs_ = 136 ± 4 min^−1^, *K*_m_(hercynine) = 436 ± 30 µM, *K*_m_(cysteine) = 603 ± 40 µM), exhibiting 62‐fold higher specificity for L‐cysteine over γ‐GC to avoid glutathione competition [124]. The N‐terminal methyltransferase domain of NcEgt1 shows 100‐fold faster rates with hercynine than histidine, underscoring substrate optimization [125]. NcEgt2 (C‐S lyase) has *k*_obs_ = 684 ± 12 min^−1^ and *K*_m_ = 195 ± 10 µM [126]. These parameters highlight the advantages of bacterial enzymes in substrate specificity but disadvantages in multi‐step complexity and lower turnover for lyases (*k*_cat_ often < 0.1 s^−1^), while fungal pathways offer higher overall efficiency through fusion and substrate shifts, though detailed kinetics for enzymes like PeEgt1 or AfEgtA remain limited [119, 124, 125]. Future efforts, including AI‐driven mutagenesis, could optimize these for industrial EGT production.

These quantitative comparisons underscore how the structural divergences translate into distinct catalytic performances across pathways. The conserved yet divergent genetic architectures supporting these enzymatic differences are summarized in Table 3, which details the key genes involved in EGT biosynthesis across bacterial and fungal species Table 3. In bacteria, such as *M. smegmatis* and *M. tuberculosis*, five genes (*egtA‐egtE*) encode enzymes for γ‐GC synthesis, C─S bond formation, glutamine amidotransfer, histidine methylation, and C─S lyase activity, respectively. In fungi, *Grifola frondosa* employs three genes (*FvEgt1*, *FvEgt2*, *FvEgt3*) for methyltransferase, cysteine desulfurase, and PLP‐dependent desulfurase functions, converting substrates like histidine, cysteine, and SAM into EGT. Similarly, *P. ostreatus* uses *PoEgt1* and *PoEgt2* for methyltransferase and desulfurase roles, while *A. fumigatus* relies on a single gene, *egtA*, for complete EGT synthesis. Other fungi, such as *N. crassa* (*NcEgt1*, *NcEgt2*) and *Trichoderma reesei* (*tregt1*, *tregt2*), utilize two‐gene systems with analogous functions. These conserved and divergent genetic components provide a foundation for efficient heterologous EGT production, enhancing yields and enabling biotechnological applications.

**TABLE 3:** Key genes involved in the ergothioneine biosynthesis.

Classification	Organisms	Genes	Function	Accession number^a^
Bacteria	*Mycobacterium smegmatis*	*EgtA*	γ‐Glutamyl cysteine synthase	WP_011731159.1
		*EgtB*	Catalyzes O_2_‐dependent C─S bond formation	WP_058127193.1
		*EgtC*	Glutamine amidotransferase	WP_058127192.1
		*EgtD*	Histidine‐specific methyltransferase	WP_058127191.1
		*EgtE*	PLP‐dependent C─S lyase	WP_058127190.1
	*Mycobacterium tuberculosis*	*EgtA*	γ‐Glutamyl cysteine synthase	NP_218221.1
		*EgtB*	Formylglycine‐generating enzyme‐like protein	NP_218220.1
		*EgtC*	Glutamine amidotransferase	NP_218219.1
		*EgtD*	Methyltransferase	NP_218218.1
		*EgtE*	Pyridoxal 5‐phosphate protein	NP_338356.1
	*Methylobacterium aquaticum*	*EgtA*	γ‐Glutamyl cysteine synthase	Maq22A_c11580
		*EgtB*	Formylglycine‐generating sulphatase	Maq22A_1p13895
		*EgtC*	Glutamine amidotransferase	Maq22A_1p31890
		*EgtD*	Methyltransferase	Maq22A_c02975
		*EgtE*	Seleno‐cysteine lyase	Maq22A_c016320
	*Chlorobium limicola*	*EanA*	Methyltransferase	—
		*EanB*	Sulfurtransferas	—
Edible fungi	*Pleurotus eryngii*	*PeEgt1*	Catalyze the formation of histidine methylation and the formation of cysteine C─S bond	—
	*Lentinula edodes*	*Lecsl*	C─S lyase	LE01Gene01404
	*Flammulina velutipes*	*Fvegtl*	Methyltransferase; hercynlcysteineteine sulfoxide synthase	MH321454.1
		*Fvegt2*	Cysteine desulfuras;	MH321455.1
		*Fvegt3*	PLP‐dependent cysteine desulfurase	MH321456.1
	*Grifola frondosa*	*GfEgt1*	SAM‐dependent methyltransferase; 5‐histidylcysteine sulfoxide synthase	OBZ71212.1
		*GfEgt2*	PLP‐dependent cysteine desulfurase	OBZ72541.1
	*Pleurotus ostreatus*	*PoEgt1*	SAM‐dependent methyltransferase; 5‐histidylcysteine sulfoxide synthase	XM_036780430.1
		*PoEgt2*	PLP‐dependent cysteine desulfurase	KDQ24319.1
Fungi	*Aspergillus fumigatus*	*egtA*	Confers complete EGT biosynthetic capacity in *Saccharomyces cerevisiae*	—
	*Neurospora crassa*	*NcEgt1*	SAM‐dependent methyltransferase; 5‐histidylcysteine sulfoxide synthase	XP_956324.3
		*NcEgt2*	PLP‐dependent cysteine desulfurase	XP_001728131.1
	*Trichoderma reesei*	*tregt1*	SAM‐dependent methyltransferase; 5‐histidylcysteine sulfoxide synthase	XM_006968558.1
		*Tregt2*	PLP‐dependent cysteine desulfurase	XM_006968673.1
	*Schizosaccharomyces pombe*	*SpEgt*1	SAM‐dependent methyltransferase; 5‐histidylcysteine sulfoxide synthase	NP_596639.2
		*SpEgt2*	PLP‐dependent cysteine desulfurase	NP_595091.1

Abbreviation: PLP: pyridoxal 5′‐phosphate; SAM: S‐adenosylmethionine.

^a^ Accession numbers of involved genes were derived from the GenBank database (https://www.ncbi.nlm.nih.gov/genbank/).

While the catalytic mechanisms of these enzymes are increasingly well‐understood, the regulation of EGT biosynthesis remains less characterized. Evidence indicates that EGT production is linked to cellular responses to oxidative stress. In mycobacteria, the clustering of EGT biosynthetic genes implies potential co‐regulation, with expression potentially induced under oxidative stress conditions to bolster cellular protection. Similarly, in fungi, EGT contributes to conidial survival and resistance to peroxide toxicity, suggesting regulatory connections to stress response pathways. However, the precise molecular mechanisms governing EGT biosynthesis regulation remain elusive, necessitating further research.

Collectively, the structural and kinetic characterization of key enzymes, such as EgtB and Egt1, reveals the catalytic complexity inherent in natural EGT synthesis. While these pathways are evolutionarily optimized, their native efficiencies are often insufficient for industrial demands due to complex metabolic regulation and suboptimal enzyme turnover. Consequently, the transition from understanding natural pathways to implementing metabolic engineering strategies is required to reprogram these biosynthetic networks for enhanced productivity.

## 4 Integrated System Engineering for Scalable and Sustainable Ergothioneine Biomanufacturing

Although EGT can be synthesized by diverse microorganisms, natural production levels are generally insufficient for industrial applications. Therefore, improving EGT production requires an integrated strategy that combines host selection, pathway reconstruction, metabolic flux optimization, enzyme engineering, cofactor balancing, and sustainable fermentation processes. These approaches collectively address major bottlenecks such as low precursor availability, intermediate toxicity, insufficient catalytic efficiency, and limited scalability, providing a route from laboratory biosynthesis to industrial biomanufacturing.

### 4.1 Host Selection and Pathway Reconstruction for High‐Yield EGT Production

**FIGURE 4:**
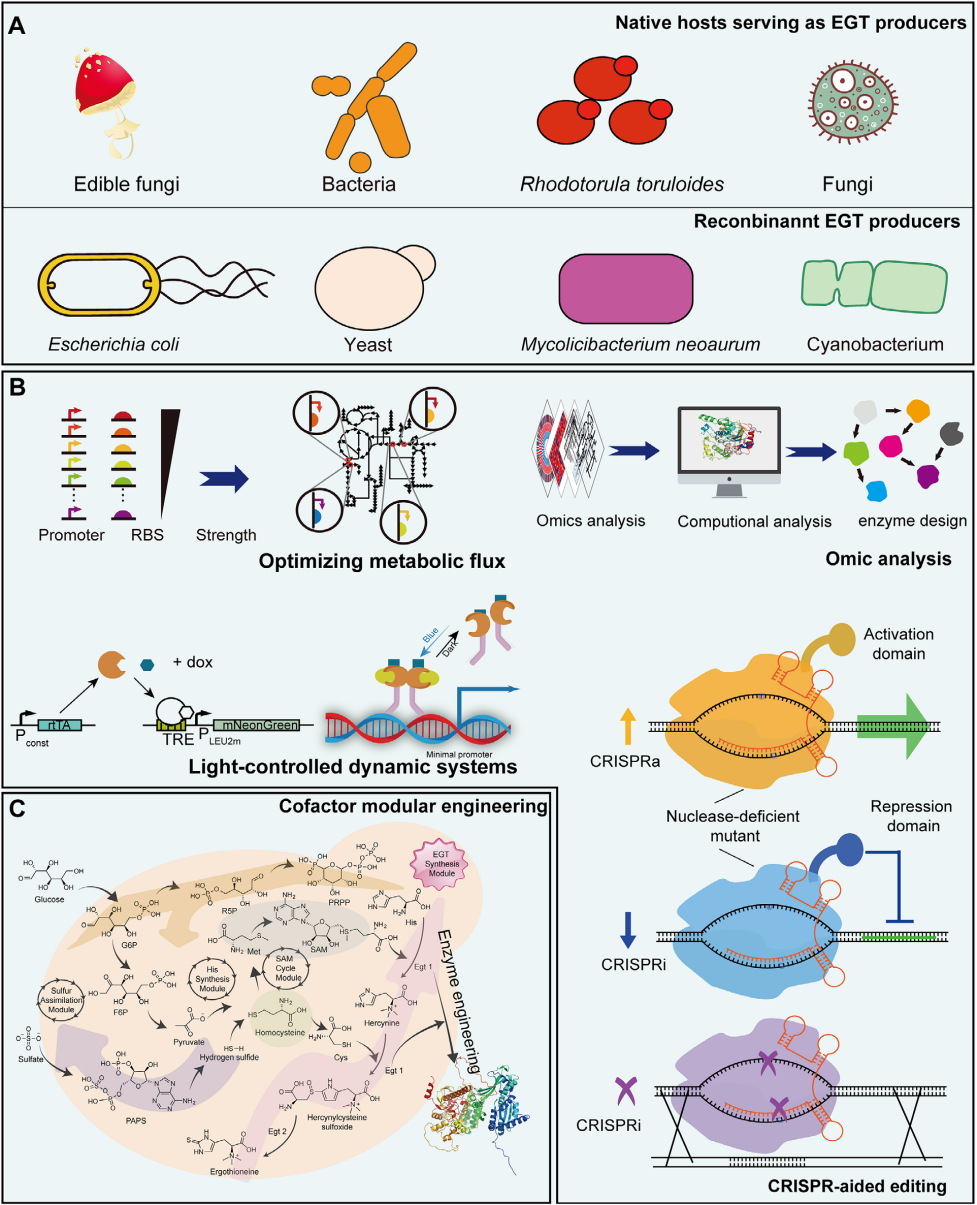
Metabolic engineering approaches to optimize EGT yield. (A) Schematic representation of wild‐type native producers and genetically engineered heterologous hosts utilized for EGT biosynthesis; (B) advanced regulatory toolkits used to optimize EGT metabolic flux, including promoter/RBS library screening for expression strength tuning, multi‐omics‐driven computational enzyme design, doxycycline‐, or light‐controlled dynamic induction systems, and CRISPR‐aided transcriptional regulation (CRISPRa/CRISPRi) or multiplexed genome editing; (C) system‐level engineering schemes illustrating the coordination of metabolic modules (glucose/sulfate assimilation, histidine, and SAM synthesis cycles) connected to the core EGT synthesis module (Egt1/Egt2), combined with structural enzyme engineering to maximize precursor availability and catalytic efficiency. RBS: ribosome binding site; dox: doxycycline; rtTA: reverse tetracycline‐controlled transactivator; TRE: tetracycline response element; CRISPRa: CRISPR activation; CRISPRi: CRISPR interference; mNeonGreen: monomeric neon green (a bright monomeric yellow‐green fluorescent protein); SAM: S‐adenosylmethionine; G6P: glucose‐6‐phosphate; F6P: fructose‐6‐phosphate; R5P: ribose‐5‐phosphate; PRPP: phosphoribosyl pyrophosphate; PAPS: 3'‐phosphoadenosine‐5'‐phosphosulfate.

Host selection is a critical determinant of EGT biosynthesis because it affects pathway compatibility, precursor availability, redox balance, genetic stability, and industrial scalability (Figure 4A).

Native producers, especially fungi, remain important sources of EGT. Basidiomycetes such as *Agaricus bisporus*, *P. citrinopileatus*, *P. eryngii*, *P. ostreatus*, *P. tuber‐regium*, *Lentinula edodes*, *Ganoderma neo‐japonicum*, *G. resinaceum*, and *Panus conchatus* exhibit variable but notable EGT productivity under optimized cultivation, precursor supplementation, light exposure, or oxidative‐stress induction conditions [127]. Yeasts and yeast‐like fungi also provide valuable production platforms, including *Rhodotorula* species, *Ustilago siamensis*, *S. pombe*, *Aureobasidium pullulans*, and *N. crassa*, with reported improvements through medium optimization, precursor feeding, and overexpression of EGT biosynthetic genes [128]. Bacterial and cyanobacterial producers, including *M. aquaticum*, *Mycolicibacterium neoaurum*, and *Oscillatoria* sp., generally show lower titers but offer unique metabolic traits for future engineering [129]. These native producers are summarized in Table 4.

**TABLE 4:** Overview of native hosts for ergothioneine production.

Native hosts	Characteristics	EGT production	Reference
*Oscillatoria sp*. CCAC M1944	Waris‐H medium (flask) for cyanobacterium with intracellular EGT	0.8–0.9 mg/g dry cell (intracellular)	[27]
*Pleurotus ostreatus*	Optimization of medium and fermentation process	> 500 mg/L	[110]
*Agaricus bisporus*	Selection of cultivation conditions and post‐harvest storage	1.3 mg/g DW (dry weight)	[127]
*Aureobasidium pullulans*	SD medium + 2% glycerol + 2% yeast extract (test tube)	14 mg/L	[129]
*Neurospora crassa*	Natural producer without precursor addition in ryan medium (flask)	0.86 mg/g dry cell	[130]
*Pleurotus citrinopileatus*	Basal medium + 2% glucose + amino acids (L‐histidine, L‐methionine, L‐cysteine)	98 mg/L	[131]
*Lentinula edodes*	Soak for fermentation in synthetic medium	0.913 mg/L	[132]
*Lentinula edodes 601*	Fructose and aspartic acid are combined to supplement L‐methionine	3.45 mg/g DW	[133]
*Lentinula edodes* L808	Blue light	2.8 mg/g DW	[134]
*Ganoderma neo‐japonicum*	Adding L‐methionine, L‐cysteine, L‐histidine	15.42 mg/L	[135]
*Ganoderma resinaceum*	Optimization of fermentation process and adding carbon and nitrogen sources	4.10 mg/L	[136]
*Pleurotus eryngii*	Optimization of fermentation process and adding L‐methionine, L‐cysteine, L‐histidine	64.2 mg/L	[137]
*Pleurotus citrinopileatus 303*	Optimization of the cultivation conditions, combining oxidative stress with clearance, first exposed to H_2_O_2_, followed by oxidative clearance using vitamin C in the second stage	641.76 mg/L	[138]
*Pleurotus tuber‐regium*	Na_2_SeO_3_‐induced oxidative stress, in combination with optimized carbon sources and precursor amino acids	35.27 mg/L	[139]
*Rhodotorula* sp. Y090	Adding glucose, glycerol, or sucrose as the sole carbon source	363.6 mg/L	[128]
*Rhodotorula mucilaginosa* DL‐X01	Optimization of fermentation process and adding three key precursor substances (histidine, methionine, and cysteine)	339.08 ± 3.31 mg/L	[140]
*Rhodotorula toruloides* 2.1389	Integrating an additional copy of EGT biosynthetic core genes *RtEGT1* and *RtEGT2* into genome, supply of S‐adenosylmethionine	267.4 mg/L	[141]
*Schizosaccharomyces pombe*	Overexpression of the gene *egt1* from *S. pombe* and *nmt1* promoter	1606.3 µM	[142]
*Ustilago siamensis CBS9960*	Culturing in YM medium and raising precursor L‐histidine	75 mg/L	[143]
*Panus conchatus*	Medium optimization (5% molasses + 3% soypeptone + 0.04% cysteine) and submerged culture	148.79 mg/L	[144]
*Mycolicibacterium neoaurum* ATCC25795	Chemically defined medium with glycerol + glucose + citric acid for actinomycete	13.3 mg/L	[145]

To overcome the limited productivity of natural hosts, heterologous expression has been widely applied in genetically tractable microbial chassis (Figure 4A and Table 5). *E. coli* has been engineered through expression of fungal or bacterial EGT pathways, precursor‐pathway reinforcement, metJ and sdaA deletions, plasmid‐copy optimization, and enzyme truncation or mutation, leading to titers ranging from the milligram‐per‐liter level to gram‐scale production [146]. Beyond *E. coli*, *Corynebacterium glutamicum* has been optimized by introducing fungal or bacterial EGT genes and strengthening cysteine assimilation [147]. Other hosts, including engineered *M. aquaticum*, *A. oryzae*, and *Cordyceps militaris*, further expand the available chassis repertoire for EGT biosynthesis [145].

**TABLE 5:** Summary of recombinant ergothioneine producers.

Heterologous host	Enzyme engineering and key strategies	EGT Yield	Quantified improvement and engineering impact	Reference
*E. coli* BW25113	Co‐exp of *M. smegmatis EgtBCDE* and *GshA*; optimization of precursor supply	24 mg/L	1.26‐fold increase via sulfur supply reinforcement	[33]
*E. coli* JW3909	Co‐exp of *egtDE* (*M. smegmatis*) and *EgtB* (*M. brachiatum*); co‐exp of *cysE*, *SerA*, and *ydeD*; Δ*metJ* to boost Met flux	657 mg/L	2.4‐fold titer boost achieved via EgtB ortholog screening and SAM/L‐Cys supply reinforcement	[119]
*C. militaris* CM15	OE of the *egtD* gene from *M. smegmatis*, truncated *egt1* and *egt2*, flask, 240 h	2.49 ± 0.05 mg/kg of media	High‐level EGT accumulation achieved by constructing a novel biosynthetic pathway	[125]
*M. aquaticum 22A*	Increasing *egtBD* copy number; Δ*HutH*; test tube, 168 h	20 mg/L	2‐fold enhancement achieved via gene copy doubling and deletion of histidine ammonia‐lyase	[129]
*S. cerevisiae EC118*	OE of *Gfegt1* and *Gfegt2* from *Grifola f rondosa*; daily addition of 1% glycerol, flask, 168 h	20.61 mg/L	1.75‐fold titer enhancement achieved by expressing a two‐gene cluster and optimizing glycerol feeding strategy	[148]
*E. coli* K12/BW25113	Co‐exp of *tregt1* and *tregt2* (*T. reesei*); fed‐batch fermentation for 143 h in a 2‐L jar fermenter	4.34 g/L	61.5‐fold increase in EGT titer via fed‐batch fermentation (from 70.59 mg/L to 4.34 g/L)	[146]
*M. neoaurum* ATCC25795	OE of the EGT synthetic gene cluster and *hisG*, *hisC*, and *allB1*, inactivated lyase gene *Mn_3042*; precursor optimization; 5‐L jar, fed‐batch, 216 h	1.56 g/L	11.1‐fold total enhancement achieved by integrating the EGT gene cluster (7.2‐fold boost), inhibiting product degradation (21% increase via *Mn_3042* knockout), and reinforcing SAM regeneration	[145]
*E. coli* JW3909	OE of *egtABCDE* from *M. smegmatis*; metabolic flux redirection towards cysteine/methionine; 3‐L jar, fed‐batch, 216 h	1.31 g/L	131‐fold titer enhancement achieved by integrating a high‐Cys production system with Met‐pathway deregulation *(met*J disruption)	[149]
*E. coli* BL21(DE3)	OE of *egtBDE* from *M. aquaticum*, serAmut and thrAmut, Δ*metJ*, Δ*sdaA*, adjusting plasmid copy numbers, enhancing precursor amino acid supply;10‐L jar, fed‐batch, 72 h	595 mg/L	17‐fold total increase, initiated by a 271.4% boost in the metabolic engineering stage via global regulator and pathway optimization	[150]
*E. coli* BL21(DE3)	Truncated, mutated *NcEgt1* from *N. crassa*, mutated *egtD* ^I50A^ from *M. smegmatis;* precursor optimization; 5‐LJar, fed‐batch, 94 h	5.4 g/L	77‐fold titer increase achieved by combining enzyme engineering (4.1‐fold boost via MD4 mutant) with optimized fed‐batch fermentation	[151]
*E. coli* BL21(DE3)	Co‐exp of *Tr1* and *Tr2* from *T. reesei*, optimizing the precursor amino acid pathways, 5‐L jar, fed‐batch, 80 h	2331.58 mg/L	11.58‐fold total increase achieved by overcoming inclusion body hurdles (2.98‐fold boost via protein engineering) and multi‐precursor module optimization (L‐Cys, L‐His, and SAM)	[152]
*E. coli* BL21(DE3)	Co‐exp of *egt1* and *egt2* ^E155C^ from *T. reesei*; membrane permeability engineering; LPS biosynthesis deletion (Δ*sdaA*, Δ*metJ*, Δ*msbB)* to increase permeability; 5‐L jar, fed‐batch, 96h	4.06 g/L	102.3‐fold total enhancement achieved by combining membrane permeability engineering (LPS deletion), catalytic capacity improvement (Tregt2^E155C^ mutant), and fermentation scale‐up	[153]
*E. coli* BL21(DE3)	N‐terminal truncation (Δ32) with mRNA‐stabilizing dtRNA elements; structure‐guided saturation mutagenesis informed by alanine scanning, ΔΔG stability predictions, MD simulations (RMSD/RMSF), and consensus design yielding six‐point mutant (G231A/E243K/N322Q/K325D/E337P/F409S) in rhodanese EanB	47.3 g/L	40.1‐fold activity increase (initial 7.7‐fold from truncation and stabilization) enabling 90.4% molar conversion in optimized in vitro chemo‐enzymatic cascade	[154]
*C. glutamicum* ET11	OE of *egt1* and *egt2* from *S. pombe*; H36 synthetic promoter for sulfur flux; Δ*sdaA*; fed‐batch in jar for 36 h	264.4 mg/L	5.9‐fold titer enhancement achieved by integrating sulfur assimilation, pentose phosphate, and L‐cysteine pathways, enabling precursor‐free de novo synthesis	[147]
*C. glutamicum* CYS‐ 2	Co‐exp of *egtB* from *M. brachiatum* and *egtDE* from *M. smegmatis;* fed‐batch in flask for 120 h	100 mg/L	2.5‐fold titer enhancement achieved by integrating an L‐cysteine‐producing host with a *M. bacterium*‐derived EgtB to optimize sulfur donor utilization	[155]
*S. cerevisiae* BY4741	Co‐exp of the gene *EGT1* from *C. purpurea* and *EGT2* from *N. crassa*, developed the IMIGE (Iterative Multi‐copy Integration by Gene Editing) system, 12 copies, 5.5–6 days	105.31 ± 1.53 mg/L	5.07‐fold titer increase achieved via the IMIGE system, enabling rapid iterative multi‐copy integration	[156]
*S. cerevisiae* SZA014	Co‐exp of *egt1* gene from *N. crassa* and *egt2* gene from *C. purpurea*, Δ*Leu2*; supplementation levels of L‐cysteine, L‐methionine, and L‐histidine,10‐ L jar, fed‐batch, 192 h.	626.24 mg/L	31.7‐fold total enhancement achieved by resolving pathway limitations through protein fusion technology (6.2‐fold flask boost)	[157]
*S. cerevisiae* CEN.PK113‐7D	Co‐exp of *egt1* from *N. crassa* and *egt2* from *C. purpurea*; 2 copies, Δ*TOR1* or Δ*YIH1*; 1‐L jar, fed‐batch, 84 h	598 ± 18 mg/L	332‐fold total enhancement achieved by resolving nitrogen metabolism bottlenecks and scaling up via systematic fed‐batch fermentation	[158]
*S. cerevisiae* CEN.PK113‐7D	Co‐exp of *egt1* from *N. crassa* and *egt2* from *C. purpurea*; 2 copies; OE of *MET14*; Δ*SPE2*; 1‐L jar, fed‐batch, 160 h	2.39 ± 0.08 g/L	22.5‐fold enhancement achieved by integrating nine validated metabolic targets and optimizing Aqr1‐mediated transport	[159]
*S. cerevisiae* IMX581	Co‐exp of *egt1* from *G. resinaceum* and *egt2* from *N. crassa*, OE of *STL1*; addition of a small amount of acetic acid, 5‐L jar, fed‐batch, 240 h	1.14 g/L	10‐fold enhancement achieved by utilizing glycerol as a non‐Crabtree‐inducing carbon source and optimizing upstream metabolism with ethanol/acetic acid supplementation	[160]
*Y. lipolytica* ST6512	Co‐exp of *egt1* from *N. crassa* and *egt2* from *C. purpurea*; 2 copies; 1‐L jar, fed‐batch, 220 h	1.63 ± 0.04 g/L	10.3‐fold enhancement achieved by doubling biosynthetic gene copies and implementing a phosphate‐limited fed‐batch strategy	[161]
*Y. lipolytica* Ku70	Co‐exp of *egt1* and *egt2* from *T. reesei*, Δ*Ku70;* strengthened the key gene *MET6* for synthesizing SAM; Δ*SPE2;* 5‐L jar, 168 h	9.3 g/L	18‐fold enhancement through protein engineering (2.41‐fold mutant boost) and four‐module precursor network optimization	[162]
*Y. lipolytica* W29	Co‐exp of *Tr1*/*Tr2*; LTR/rDNA‐mediated multi‐copy integration; Cre‐loxP iterative editing; enzyme tag and codon optimization, 5‐L jar, fed‐batch	7.3 g/L	The highest de novo L‐ergothioneine titer from glucose reported to date for a 5 L fermenter	[163]
*A. oryzae* NSAR1	OE of the *egt1* and *egt2* from *N. crassa*	231 mg/kg of media	20‐fold increase in EGT production by heterologous expression	[164]

Abbreviations: Co‐exp: co‐expression; OE: overexpression; Δ: knockout.

### 4.2 Metabolic Flux and Cofactor Engineering for Balanced Biosynthesis

Efficient EGT biosynthesis depends not only on pathway expression but also on the coordinated allocation of carbon, sulfur, nitrogen, and methyl‐group metabolism. Modular pathway design can reduce toxic intermediate accumulation and improve pathway efficiency. For example, dividing EGT biosynthesis into precursor‐supply and oxidative‐coupling modules improved production in *Y. lipolytica*, while synthetic protein scaffolds in *E. coli* enhanced local enzyme colocalization and increased EGT yield [161]. Similar strategies, including deletion of competing pathways and reinforcement of precursor flux, improved EGT production in *E. coli*, *S. cerevisiae*, and *C. glutamicum* [150]. Redirecting metabolic flux toward histidine, cysteine, methionine, and SAM supply is a central strategy for improving EGT yield (Figure 4B,C). In *E. coli*, deletion of *metJ* and overexpression or mutation of genes involved in cysteine, serine, and SAM metabolism enhanced precursor availability and increased production [119]. In *S. cerevisiae*, deletion of SAM‐consuming pathways, improvement of glycerol utilization, and CRISPR‐Cas9‐mediated pathway integration markedly increased EGT titers [159, 160]. Dynamic regulation systems, including light‐controlled expression, chemical induction, CRISPR‐Cas‐mediated integration, and CRISPRi‐based control, further enable temporal balancing between cell growth and product formation, although their scalability in high‐density fermentation (HDF) still requires optimization [165].

SAM is particularly important because it provides the methyl donor for EgtD‐mediated histidine trimethylation to hercynine. Increasing SAM biosynthesis through metK overexpression, methionine‐cycle reinforcement, and improved precursor supply has significantly enhanced EGT production in *M. neoaurum*, *E. coli*, *R. toruloides*, *Y. lipolytica*, and *K. lactis* [145]. Broader precursor engineering, including histidine, cysteine, methionine, PPP‐related flux, and glycerol assimilation, further supports SAM regeneration and improves overall pathway efficiency [145].

Metabolic engineering has improved EGT production by increasing precursor supply, optimizing methyl‐donor availability, and reducing pathway bottlenecks. For biomedical research, the practical value of these approaches lies in providing reproducible and high‐purity EGT for mechanistic studies, animal experiments, and early translational evaluation. While these pathway‐level optimizations lay a strong foundation for high‐titer production, the catalytic performance of the key biosynthetic enzymes frequently remains the ultimate rate‐limiting factor. Therefore, targeted enhancement of intrinsic enzyme properties can deliver substantial further improvements in yield and process economics.

### 4.3 Enzyme Engineering to Overcome Catalytic Bottlenecks

Although metabolic engineering improves precursor supply and pathway flux, the catalytic properties of key biosynthetic enzymes often remain limiting. EgtB, which catalyzes oxygen‐dependent C─S bond formation between γ‐glutamylcysteine and hercynine, is a major engineering target [166]. Structural studies of EgtB have revealed a distinctive iron‐centered catalytic site, substrate‐orienting residues, oxygen‐activation steps, and the importance of Tyr377 in directing sulfoxide formations [167]. The iron center adopts octahedral coordination, involving three histidine residues (His51, His134, and His138) from the enzyme, the thiolate of γGC, the imidazole nitrogen of TMH, and a water molecule, which is likely displaced by O_2_ during catalysis [167, 168]. Cryo‐EM and computational analyses further showed that conformational dynamics around the iron center and substrate‐binding pockets regulate substrate positioning, O_2_ and product release [169].

**FIGURE 5:**
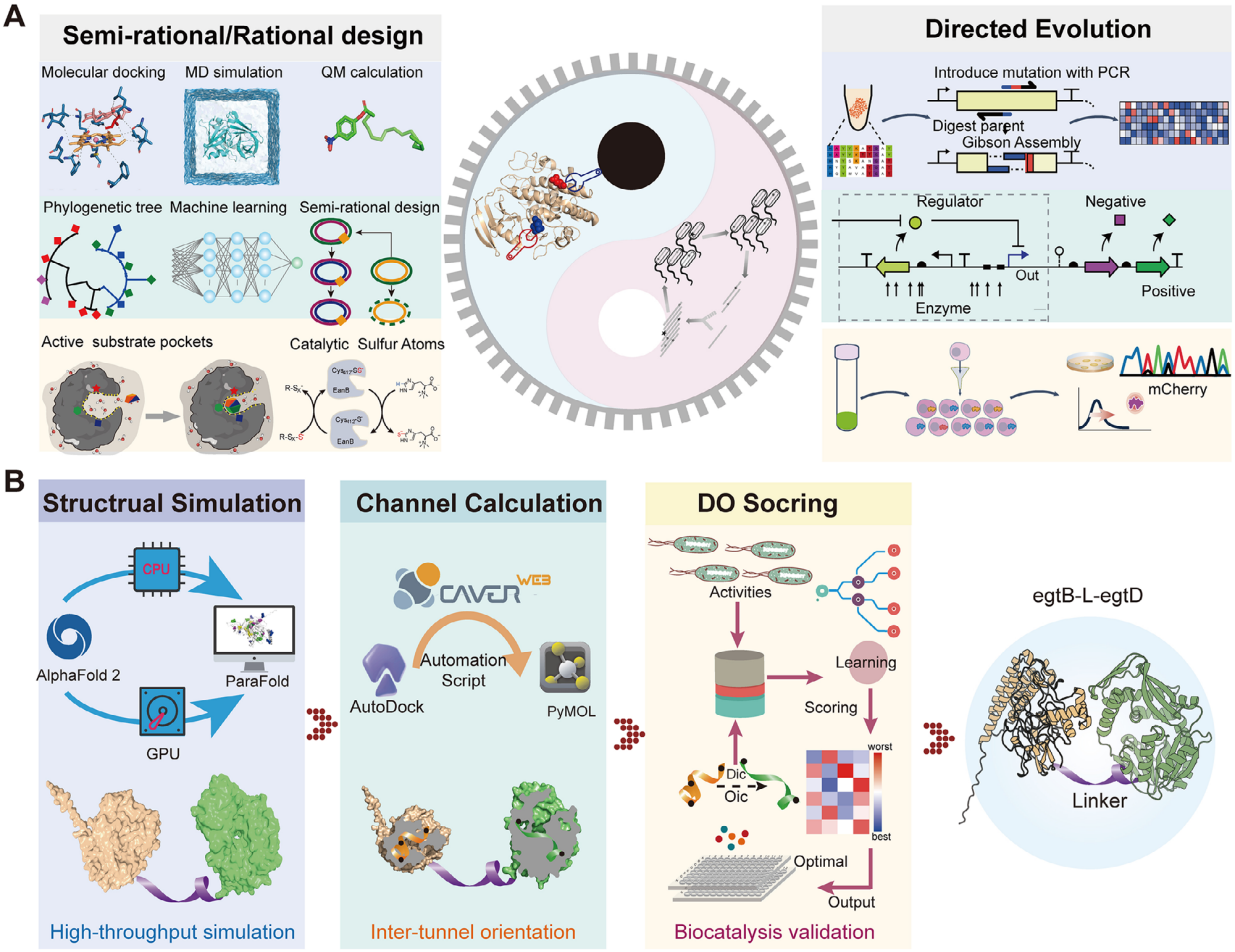
Enzyme engineering for enhanced catalytic efficiency. (A) A schematic overview contrasts semi‐rational or rational design with directed evolution as complementary methodologies for single‐enzyme optimization, where rational and semi‐rational approaches leverage structural biology and computational tools, including molecular docking, MD simulations, QM calculations, phylogenetic analysis, and machine learning, to target active substrate pockets and key catalytic residues, while directed evolution concurrently drives laboratory‐scale adaptation through random mutagenesis, Gibson assembly, high‐throughput screening, and biosensor‐based selection; (B) A high‐throughput computational workflow for multi‐enzyme fusion complexes outlines a step‐by‐step pipeline for designing and validating multi‐enzyme chimeras, which progresses from CPU/GPU‐accelerated structural prediction to substrate channel/tunnel calculation, and concludes with automated scoring and machine learning validation to identify optimal biocatalytic linker configurations. MD: molecular dynamics; QM: quantum mechanics; PCR: polymerase chain reaction; CPU: central processing unit; GPU: graphics processing unit.

These mechanistic insights have enabled rational and semi‐rational engineering of EGT biosynthetic enzymes. Structure‐guided mutagenesis, molecular docking, molecular dynamics, QM/MM simulations, and active‐site redesign have been used to improve substrate affinity, selectivity, stability, and catalytic efficiency (Figure 5A) [139, 168, 170]. Engineered EgtB variants and mutated or truncated Egt1 enzymes have improved EGT production, while error‐prone PCR and saturation mutagenesis generated high‐performing mutants that supported gram‐scale fermentation [166]. More recently, engineered anaerobic EanB enabled a chemo‐enzymatic cascade that produced 47.3 g/L EGT with high molar conversion, highlighting the potential of noncanonical sulfurtransferases for scalable EGT synthesis [154].

Directed evolution and artificial enzyme design further expand the engineering toolbox. Random mutagenesis, UV/lithium chloride mutagenesis, and combined rational‐random strategies have produced improved strains and enzymes, including TNcEgt1/EgtD‐derived mutants, high‐yield *S. pombe* mutants, and engineered *E. coli* strains [151]. Chimeric and fusion enzymes can shorten the biosynthetic route and reduce intermediate diffusion, as shown by fusion of truncated NcEgt1 with CtEgtB, co‐expression of *T. reesei* enzymes, and protein‐fusion strategies in yeast [146, 157, 165]. Computational design tools such as iMARS integrate linker databases, protein‐structure prediction, docking, channel analysis, and activity scoring to support artificial multi‐enzyme complex design (Figure 5B) [171].

Enzyme engineering may also broaden product scope, as fungal Egt1 can participate in selenoneine formation under selenium‐supplemented conditions [142]. In addition, cofactor‐oriented enzyme engineering remains important: SAM directly supports methylation, while NADPH indirectly contributes to cysteine and histidine precursor synthesis; therefore, optimization of SAM, NADPH, and related feedback‐control systems can further improve EGT biosynthesis [118]. To date, metabolic engineering and enzyme engineering have significantly propelled EGT titers in various microbial platforms to gram‐scale levels. However, achieving high productivity in laboratory‐scale fermenters is only the first step toward commercialization. To ensure economic and environmental viability, these engineered strains must be integrated into advanced industrial bioprocessing frameworks, where HDF and sustainable feedstock utilization become the primary considerations for large‐scale translation.

### 4.4 Industrial Scale‐Up and Sustainable Production Platforms

The industrial translation of EGT is driven by expanding demand in pharmaceutical, nutraceutical, cosmetic, and functional food sectors. The global market has grown rapidly, with projected continued expansion, supported by advances in synthetic biology and fermentation technology [172]. Industrial progress is already evident in cosmetic applications, where fungal co‐fermentation has enabled high‐purity EGT production and reduced manufacturing costs [38]. In nutraceuticals and functional foods, commercial EGT ingredients and supplements have expanded following GRAS notification by the US FDA and Novel Food approval by EFSA, with clinical studies supporting potential benefits for sleep quality, cognitive health, and neurodegeneration‐related biomarkers [173].

**FIGURE 6:**
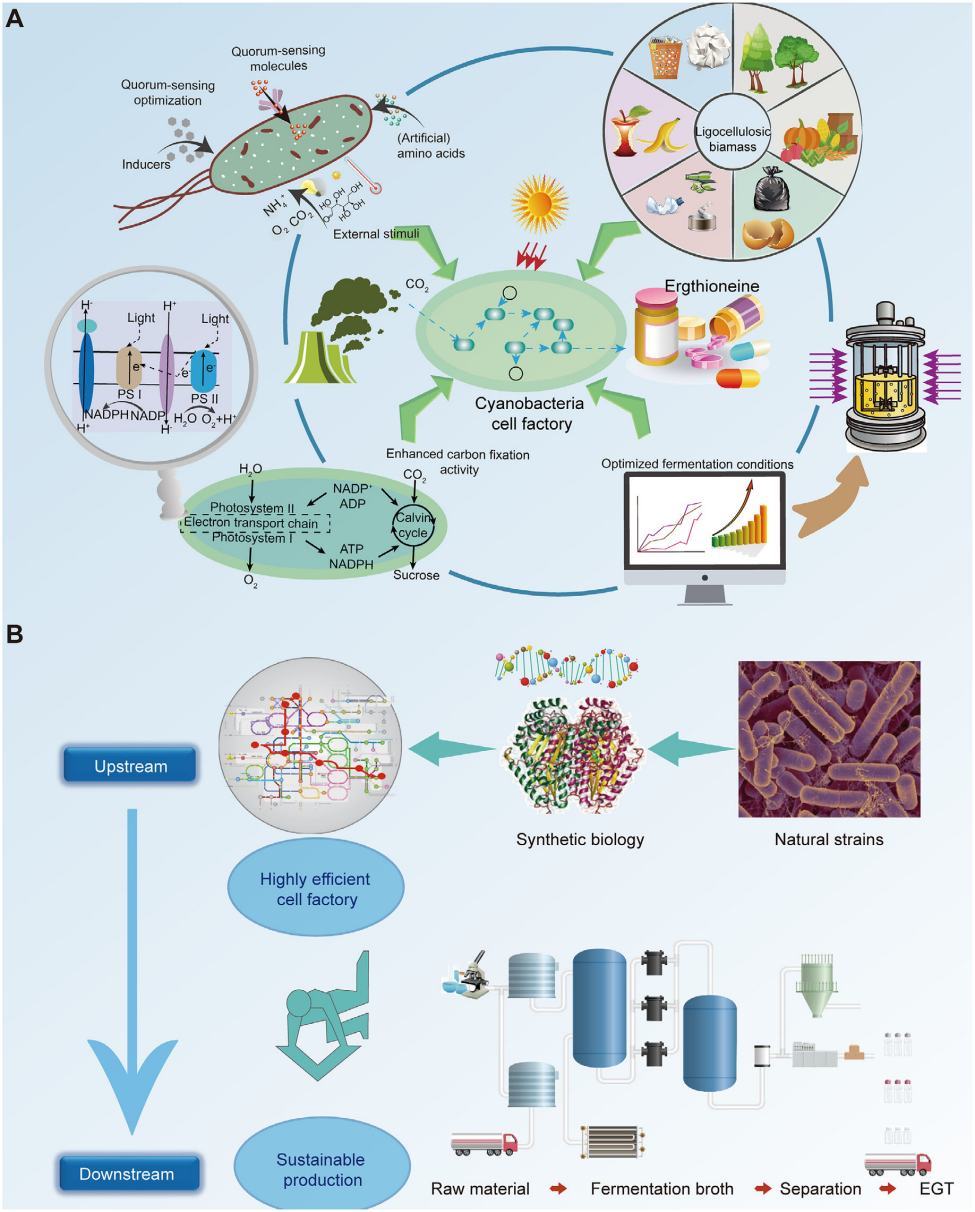
Industrial biotransformation and sustainable production. (A) A multi‐dimensional regulation strategy for establishing carbon‐neutral cell factories provides a schematic representation of integrated bioprocess optimization centering on a cyanobacteria cell factory, where the system leverages renewable inputs like lignocellulosic biomass and external chemical stimuli alongside solar‐driven carbon fixation. Enhanced photosynthetic electron transport (Photosystem I and II) couples energy generation directly to the calvin cycle for efficient CO_2_ assimilation, which is further scale‐up integrated via optimized fermentation monitoring and bioreactor control systems to maximize EGT yield. (B) An integrated upstream‐to‐downstream manufacturing pipeline presents an integrated workflow spanning from genetic engineering to industrial purification, in which the upstream stage focuses on developing highly efficient cell factories through synthetic biology remodeling of natural wild‐type strains, while the downstream stage establishes a sustainable, closed‐loop pipeline for large‐scale production by transitioning sequentially from raw material input and bioreactor fermentation broth processing to high‐resolution separation, purification, and final formulation of pure EGT. PS I: Photosystem I; PS II: Photosystem II; ATP: adenosine triphosphate; ADP: adenosine diphosphate; NADPH: nicotinamide adenine dinucleotide phosphate (reduced); NADP^+^: nicotinamide adenine dinucleotide phosphate (oxidized).

To sustain this market expansion, EGT production must move beyond laboratory titers toward economically viable and environmentally sustainable processes. HDF is central to this transition because it increases volumetric productivity and reduces space and resource requirements (Figure 6A). Quorum sensing (QS)‐based regulation may provide autonomous, inducer‐free control of EGT biosynthetic pathways by coupling gene expression to cell density and metabolic state [174, 175]. Although direct QS regulation of EGT remains underexplored, related studies in *M. smegmatis* suggest that QS‐associated signals such as c‐di‐GMP may influence stress responses, biofilm formation, and redox balance, which are relevant to EGT biosynthesis [176]. Similar QS circuits have improved carbon‐flux allocation in other bioprocesses and may be adapted to regulate precursor supply and reduce metabolic imbalance during HDF [174, 175].

Sustainable feedstocks are another key requirement for industrial EGT production. Industrial glycerol and lignocellulosic biomass offer low‐cost, renewable alternatives to refined glucose. Glycerol supports aerobic growth of *S. cerevisiae* and *E. coli*, can reduce Crabtree‐effect‐related limitations, and has already been applied in high‐yield EGT production systems [177]. However, slower glycerol uptake and impurities in crude glycerol require further strain engineering, improved glycerol kinase activity, tolerant hosts, or pretreatment strategies [178].

Lignocellulosic hydrolysates could provide fermentable sugars for EGT production, although direct EGT production from lignocellulose remains insufficiently explored; inhibitor‐tolerant strains and detoxification methods may be required to overcome the effects of furfural, acetic acid, and related inhibitors [159, 179]. Fed‐batch and continuous fermentation strategies, together with optimized nutrient feeding, pH, temperature, and oxygen control, will be essential for integrating these substrates into scalable EGT manufacturing [151].

Light‐driven and carbon‐fixing platforms offer a longer‐term route toward carbon‐negative EGT production. Cyanobacteria can fix atmospheric CO_2_ through the Calvin–Benson–Bassham cycle and naturally accumulate EGT or its biosynthetic intermediate hercynine, supporting their potential as sustainable production chassis [180, 181, 182]. Coupling CO_2_ fixation with EGT biosynthesis may channel photosynthetically fixed carbon into histidine and cysteine precursors, while EGT itself may help counteract photosynthesis‐associated ROS stress [183]. Engineering strategies, including overexpression of native or heterologous EGT genes, CRISPR‐Cas9‐mediated regulation, precursor‐pathway optimization, light‐condition tuning, and genome‐scale metabolic modeling, could improve cyanobacterial EGT production while maintaining carbon‐fixation efficiency [149].

In practice, no single production platform is likely to meet all research and translational needs. For near‐term biomedical studies, microbial fermentation and optimized heterologous expression remain the most practical routes. More exploratory systems, such as cyanobacterial or carbon‐fixing platforms, may become useful later but should currently be viewed as early‐stage approaches (Figure 6B) [184]. The integration of advanced fermentation control, sustainable feedstocks, and carbon‐negative synthesis via cyanobacterial systems offers a robust framework for the green manufacturing of EGT. These technological advancements ensure that the burgeoning demand for EGT can be met without compromising environmental integrity. Ultimately, the synthesis of these industrial strategies, combined with emerging computational tools, paves the way for the next generation of EGT‐based therapeutics and health products.

## 5 Concluding Remarks and Future Perspectives

EGT has attracted increasing attention as a diet‐derived sulfur‐containing compound with antioxidant, anti‐inflammatory, and cytoprotective properties. Unlike many small‐molecule antioxidants, its biological distribution is strongly influenced by OCTN1‐mediated uptake and tissue retention. This transporter‐dependent feature provides an important basis for understanding why EGT preferentially accumulates in metabolically active or stress‐sensitive tissues and why its effects may vary across physiological and pathological contexts.

Current evidence suggests that EGT may be relevant to a range of oxidative stress‐ and inflammation‐related conditions, including neurodegenerative, cardiovascular, metabolic, renal, hepatic, intestinal, and age‐associated disorders. Mechanistically, EGT has been linked to ROS/RNS scavenging, metal ion chelation, mitochondrial protection, Nrf2 activation, NF‐κB inhibition, regulation of inflammatory cascades, and modulation of cellular senescence‐related pathways. These findings provide a plausible biological rationale for its protective effects observed in preclinical models. However, most disease‐related evidence remains experimental, and the available human studies are still limited by small sample sizes, short intervention periods, and exploratory endpoints. Therefore, the clinical value of EGT should be interpreted cautiously until larger, longer‐term, and disease‐specific randomized controlled trials are available.

A central message emerging from this review is that OCTN1 is not simply a passive transporter of EGT, but a key determinant of its tissue exposure and biological activity. In aging and neurodegenerative settings, OCTN1‐dependent EGT accumulation may support redox homeostasis, mitochondrial function, and resistance to stress‐induced cellular damage. In tumor biology, however, the OCTN1/EGT axis may have more complex and context‐dependent effects. While EGT may protect normal tissues from oxidative injury, increased OCTN1‐mediated EGT uptake in certain tumor cells could theoretically enhance antioxidant capacity and help malignant cells tolerate metabolic or therapeutic stress. Future studies should therefore evaluate OCTN1 expression, EGT accumulation, tumor type, disease stage, and treatment context before drawing conclusions about the safety or benefit of EGT supplementation in cancer‐related settings [185].

From a translational perspective, reliable access to high‐purity EGT remains important for mechanistic studies, animal experiments, and future clinical research. Microbial biosynthesis, enzymatic production, and metabolic engineering approaches can help provide stable and scalable sources of EGT. Nevertheless, in a medically oriented review, these production strategies should be viewed primarily as enabling tools rather than as the central focus. Their practical value lies in supporting reproducible biomedical research, improving compound availability, and facilitating well‐controlled preclinical and clinical investigations.

**FIGURE 7:**
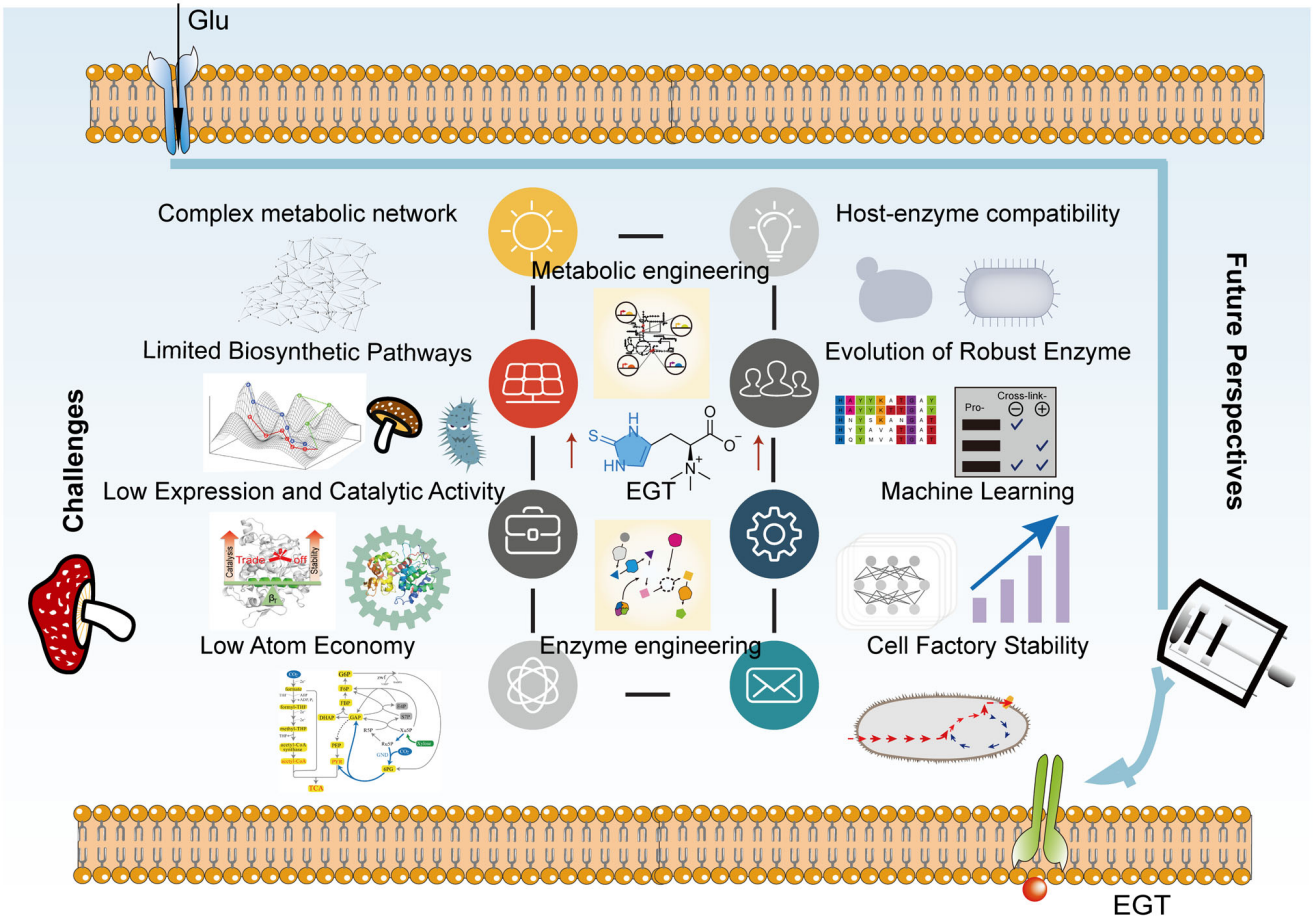
Current challenges and prospective development strategies for the sustainable biomanufacturing of EGT. A framework delineates the transition from bottleneck mitigation to advanced synthetic biology solutions, where current technological challenges, including complex metabolic networks, limited biosynthetic pathways, low enzyme expression and catalytic activity, and poor atom economy, are strategically addressed through multi‐dimensional metabolic and enzyme engineering frameworks. Looking forward, future perspectives emphasize host‐enzyme compatibility refinement, the evolution of robust enzymes, and machine learning‐driven optimization pipelines to collectively reinforce cell factory stability, thereby accelerating the industrial‐scale bioproduction and efficient efflux of high‐purity EGT.

Several priorities should guide future research. First, clinical studies should move beyond general safety evaluation and include clearly defined disease populations, adequate sample sizes, longer follow‐up, and clinically meaningful endpoints. Second, OCTN1 should be incorporated as a key biological variable, including its expression, genetic variation, tissue distribution, and disease‐specific regulation. Third, more attention should be paid to the possible dual effects of EGT in aging and tumor biology, especially in contexts where antioxidant supplementation may have different consequences in normal and malignant tissues. Fourth, standardized EGT preparations, dosing regimens, pharmacokinetic assessments, and biomarker panels are needed to improve comparability across studies. Finally, production technologies should continue to be optimized to provide high‐quality EGT for biomedical use, while avoiding an overemphasis on industrial translation at the expense of mechanistic and clinical relevance. By integrating emerging technologies with solutions to existing technical and industrial barriers, EGT production systems are poised to become more sustainable and versatile (Figure 7). This progress will effectively meet the rising global demand for this valuable antioxidant, ultimately enhancing human health and well‐being.

Future EGT research should also benefit from closer integration between biomedical validation and enabling synthetic biology. On the biomedical side, greater attention should be given to OCTN1/ETT (The EGT transporter, ETT)‐mediated transport, tissue‐specific accumulation, and related sulfur‐ or selenium‐containing histidine derivatives, because these factors may help explain why EGT shows context‐dependent effects in oxidative stress, inflammation, mitochondrial function, and disease susceptibility [186, 187, 188, 189, 190]. On the production side, recent progress in microbial biosynthesis suggests that EGT cell factories can be further improved by reconstructing methyl‐ and sulfur‐donor supply, expanding biosynthetic enzyme discovery, and combining CRISPR‐based genome editing with systems‐level metabolic modeling [191, 192, 193, 194, 195, 196]. In particular, next‐generation base editing, prime editing, enzyme‐constrained genome‐scale models, and AI‐assisted structure prediction may provide useful tools for optimizing precursor flux, enzyme compatibility, pathway balance, and transporter/export modules in future EGT‐producing strains [185, 197, 198, 199, 200, 201]. These approaches may help connect scalable production with reproducible biomedical studies, thereby supporting better‐defined preclinical experiments and more reliable clinical translation.

In summary, EGT represents a biologically intriguing molecule at the intersection of diet‐derived cytoprotection, OCTN1‐mediated transport, redox biology, aging, and disease modulation. Its therapeutic potential is promising but not yet fully established. A more balanced research framework, integrating transporter biology, disease‐specific mechanisms, rigorous clinical validation, and reliable production methods, will be essential for clarifying the true biomedical value of EGT.

## Author Contributions

G.Y. drafted the manuscript. G.Y., G.L., and M.L. revised the manuscript. G.Y. and M.L. visualized the data. G.Y., D.Y., C.D., and X.T. investigated the study. G.L., M.L., and Y.X. supervised the study. M.L. and Y.X. conceptualized the study. Y.X. acquired funding. All authors reviewed the manuscript.

## Ethics Statement

The authors have nothing to report.

## Conflicts of Interest

Yongmei Xie is an employee in Chengdu Chuanyu Jianwei Biotechnology Co. Ltd. The other authors declare no conflicts of interest.

## Data Availability

The authors have nothing to report.
